# Recent Advances in Sustainable Single‐Atom Catalysts from Biomass and Solid Waste: Design, Synthesis and Applications

**DOI:** 10.1002/advs.202524310

**Published:** 2026-03-13

**Authors:** Hongzhe He, Yuhang Qiu, Ruoqun Zhang, Sasha Yang, Jingwei Wang, Jianglong Yu, Lian Zhang, Ning Chen, Baiqian Dai

**Affiliations:** ^1^ Department of Chemical and Biological Engineering Monash University Melbourne Victoria Australia; ^2^ Suzhou Industry Park Monash Suzhou Research Institute Suzhou Industry Park Suzhou China; ^3^ School of Science Jimei University Xiamen China; ^4^ Innovation Center for Chemical Sciences College of Chemistry Chemical Engineering and Materials Science Soochow University Suzhou Jiangsu China; ^5^ State Key Laboratory of Radiation Medicine and Protection Soochow University Suzhou Jiangsu China

**Keywords:** biomass‐derived, electrocatalysts, single atom catalysts, solid waste valorization

## Abstract

Single‐atom catalysts (SACs), featuring isolated metal atoms anchored on engineered supports, have emerged as a transformative class of catalytic materials. Their atomically dispersed active sites enable nearly 100% metal utilization, unique electronic structures, and highly tunable coordination environments, which results in exceptional activity and selectivity across diverse reactions. Recently, sustainable single‐atom catalysts (Sus‐SACs) derived from biomass and solid waste have garnered increasing attention. The development of Sus‐SACs not only provides a low‐cost and renewable pathway for SAC fabrication but also aligns with global goals of carbon neutrality and the circular economy. This review presents a comprehensive overview of the current progress in Sus‐SACs. First, the typical biomass‐ and solid waste‐derived precursors are introduced. The synthesis methodologies are thoroughly discussed, with particular emphasis on ultrafast synthesis approaches that hold promise for scalable production. State‐of‐the‐art techniques for structural characterization of single‐atom sites are further summarized, alongside the introdcution of emerging applications of artifical intelligence in the rapid precursor screening, design, synthesis and characterizations of Sus‐SACs. Recent catalytic applications in electrocatalysis, chemical synthesis and upgrading, and environmental remediation are systematically evaluated. Finally, the key challenges and future opportunities are outlined to guide the continued advancement and industrial translation of Sus‐SACs. Overall, this review aims to provide a comprehensive summary of developments in this rapidly evolving field and to offer insights for addressing the critical challenges that remain.

## Graphical abstract

Biomass‐ and solid waste‐derived sustainable single‐atom catalysts (Sus‐SACs) provide a cost‐effective and renewable approach to catalyst design. This review summarizes precursor selection, including AI‐assisted screening, synthesis strategies with emphasis on ultrafast methods, and advanced characterization techniques. Recent applications in electrocatalysis, chemical conversion, and environmental remediation are discussed, together with key challenges and directions for scalable development.

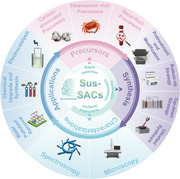

## 1 Introduction

Catalysis underpins the vast majority of modern industrial production, making the development of efficient and robust catalysts a continual priority. High‐performance catalysts facilitate reactions with elevated activity and selectivity, thereby enhancing energy efficiency and reducing emissions across diverse industrial sectors [1]. Conventional heterogeneous catalysts typically comprise metal particles of wide distribution sizes [2]. However, only those within a narrow, favorable size range contribute meaningfully to catalytic activity, while particles outside this range tend to be inactive or facilitate undesirable side reactions. Moreover, the atom utilization ratio of these catalysts is low, and reaction selectivity is normally poor, resulting in high operation cost. Furthermore, these catalysts typically suffer from low metal atom utilization and limited reaction selectivity, which in turn increases operational costs [3]. Homogeneous catalysts with well‐defined active sites, on the contrary, enables excellent activity and selectiv. However, their operational stability and recyclability remain major challenges, limiting their broad application [4].

Single‐atom catalysts (SACs), consisting of isolated metal atoms anchored on a support material, have recently emerged as a cutting‐edge class of materials and exhibit remarkable catalytic performance across a wide range of reactions [5]. Reducing metal species to the atomic scale imparts several key advantages, including altered electronic structures, highly unsaturated coordination environments, strengthened metal‐support interactions, and nearly 100% utilization of metal atoms [6]. Therefore, SACs integrates the merits of both homogeneous and heterogeneous catalysts. The rational design of SACs relies fundamentally on the precise selection of metal single‐atom centers and the engineering of suitable supports. The electronic configuration, redox properties, and intrinsic catalytic preferences of the chosen metal significantly influence catalytic performance. Equally critical is the support design. The strong metal‐support interactions not only prevent aggregation but also modulate the geometric and electronic structure of the active sites, thereby enabling tailored adsorption of reaction intermediates and synergistic catalytic performance between SAs and support [7]. Typical supports for SACs synthesis include metal oxides, metal organic frameworks, metal alloy and carbon materials. Among which, carbon materials are cost‐effective and promising for anchoring metal atoms due to their high chemical stability, tunable morphology, abundant surface functional groups and defects, and excellent conductivity (for electrocatalysis) [8].

The use of biomass and solid waste as precursors for synthesizing SACs represents a sustainable and resource‐efficient strategy [9]. Developing sustainable SACs (Sus‐SACs) aligns closely with global initiatives aimed at carbon neutrality and the advancement of a circular economy. According to statistical estimates, global biomass residues are produced at a rate of approximately 140 Gt per year, whereas global solid‐waste generation is around 2.2 Gt annually [10, 11]. On one hand, the large scale of this waste stream presents significant challenges for disposal and contributes to environmental pollution. On the other hand, these biomass and solid‐waste resources contain abundant recyclable components, offering substantial potential for valorization. Converting such waste into Sus‐SACs not only mitigates the environmental burden but also transforms discarded materials into valuable functional catalysts, effectively turning waste into wealth. Biomass, which is rich in carbon and often contains abundant heteroatoms, can be easily converted into carbon materials with diverse morphologies and structures capable of strongly stabilizing isolated metal atoms [12, 13]. Meanwhile, solid waste, such as municipal sewage and plastic products, not only provides abundant carbonaceous matrices but also contains intrinsic metal contents that can be used as a metal source for SACs synthesis [14].

**SCHEME 1:**
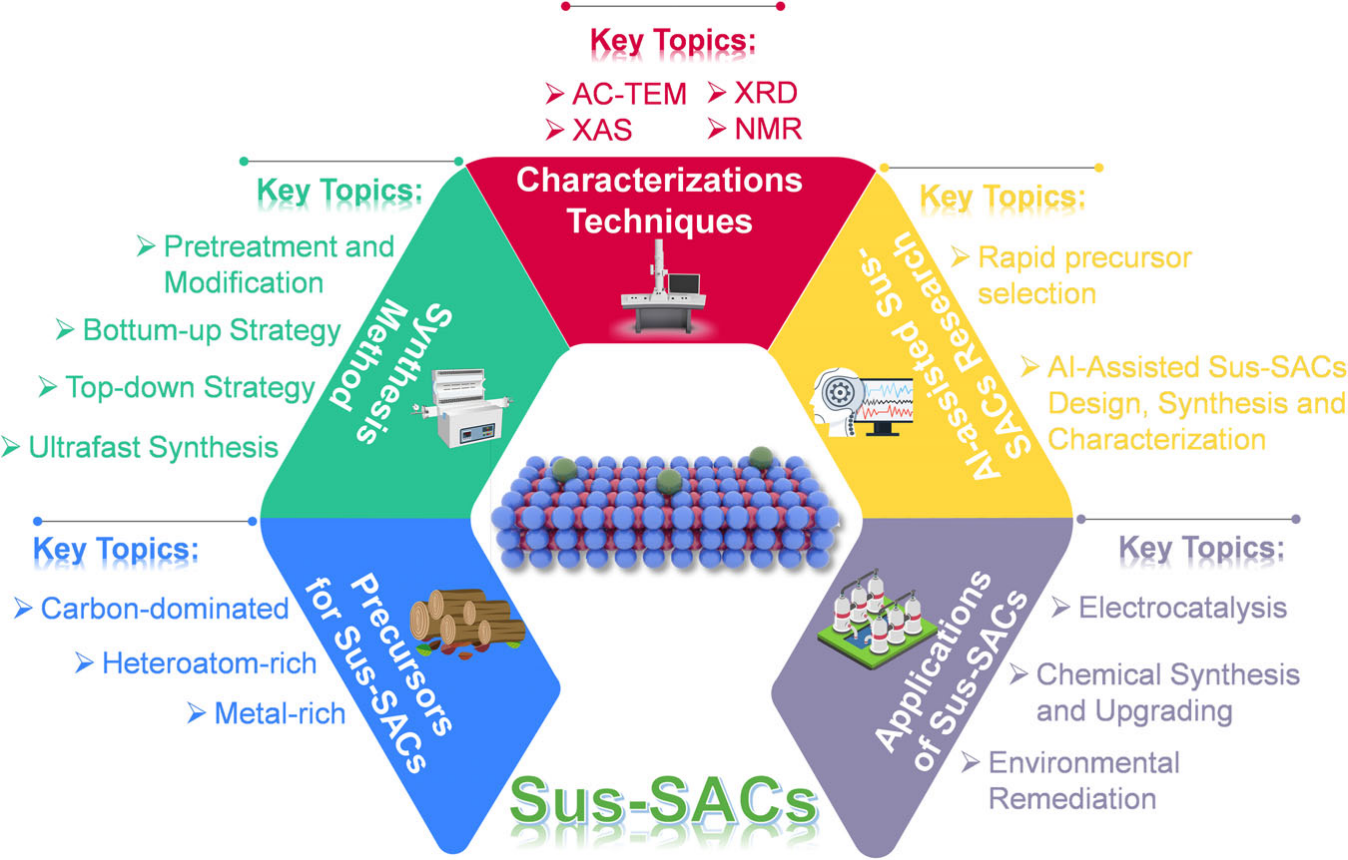
Overview of the review scope and structure.

Since its emergence in 2018, the field ofSus‐SACs has experienced rapid growth. Although several comprehensive reviews have summarized recent advances, none have systematically covered SACs derived from solid‐waste precursors, leaving an important gap in understanding the full landscape of sustainable feedstocks [15, 16, 17]. Meanwhile, the rise of artificial intelligence (AI) offers transformative opportunities for this field. For example, AI‐assisted rapid spectroscopic analysis enables accelerated precursor identification and screening, greatly shortening the development cycle. In addition, most reported Sus‐SACs are fabricated via pyrolysis, a time‐ and energy‐intensive process that poses challenges for large‐scale production. The recent emergence of ultrafast synthesis strategies provides a promising solution, offering the potential to significantly accelerate manufacturing and advance the industrial deployment of Sus‐SACs. This review also summarizes the latest advances in Sus‐SACs for electrocatalysis, chemical synthesis and upgrading, and water pollution treatment. Finally, we outline the key challenges and future opportunities in this rapidly evolving field, aiming to provide insights and guidance for the continued development ofSus‐SACs. The scope and structure of this review are presented in Scheme 1.

## 2 Precursors for Sus‐SACs Synthesis

**FIGURE 1:**
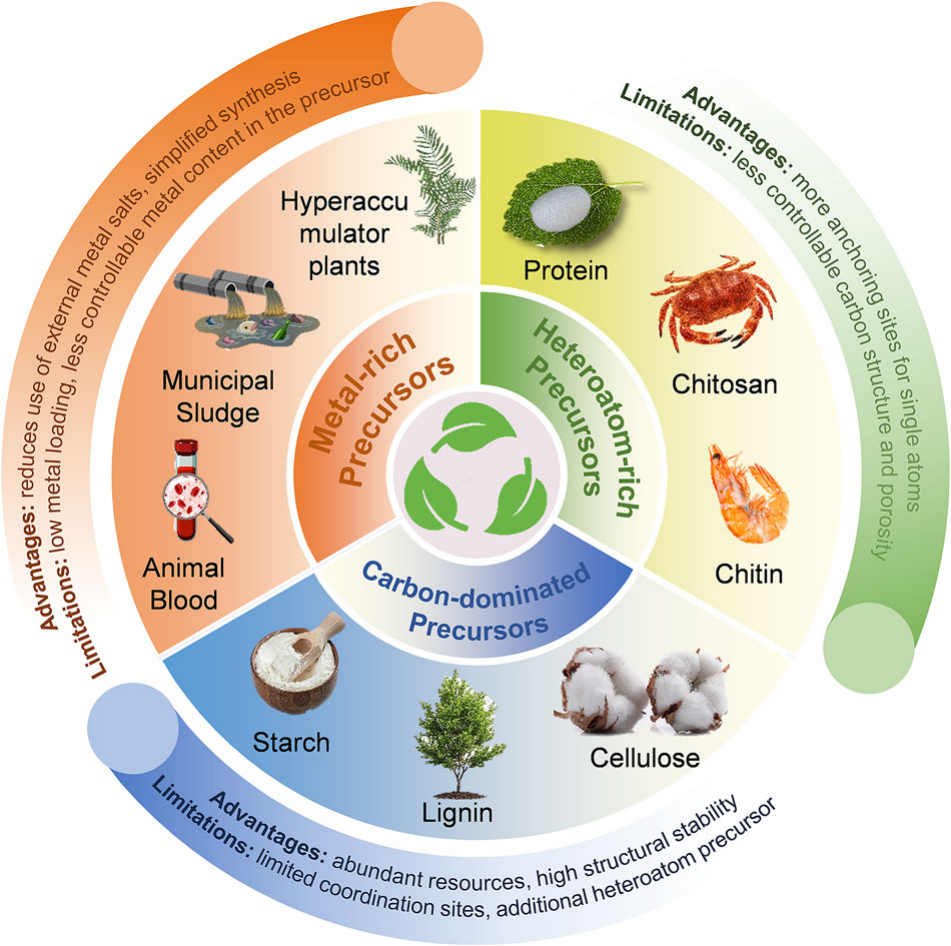
Classification of precursors for Sus‐SAC synthesis, highlighting representative examples along with their advantages and limitations.

The catalytic performance and stability of Sus‐SACs depend on the catalyst structure, coordination environment of the single atom [18]. Therefore, the selection and rational design of precursors play a pivotal role in synthesizing high‐performance Sus‐SACs. Biomass and solid waste, as renewable and widely available resources, offer a sustainable and versatile platform for Sus‐SAC synthesis [19]. Biomass materials, owing to their intrinsic hierarchical architectures, abundant functional groups, and high heteroatom content (e.g., N, S, P), are ideal candidates for constructing robust carbonaceous supports, creating a controllable coordination environment, and inhibiting single‐atom agglomeration [20]. Meanwhile, certain biomass sources and solid wastes contain high concentrations of metal elements and/or carbonaceous fractions which can simultaneously supply both the metal center and the carbon support [21]. Based on these characteristics, in this section, typical biomass and solid waste precursors for Sus‐SACs synthesis are introduced according to the following categories: (i) carbon‐dominated precursors; (ii) heteroatom‐rich precursors, (iii) metal‐rich precursors. Figure 1 summarizes representative precursors within each category, along with their corresponding advantages and limitations, providing a structured overview to guide precursor selection for the rational design of Sus‐SACs.

### 2.1 Carbon‐Dominated Precursors

Herein, carbon‐dominated biomass precursors refer to biomass or waste resources with high carbon content but intrinsically low levels of functional heteroatoms such as nitrogen, sulfur, phosphorus etc. Typical examples include lignocellulosic materials (e.g., cellulose, lignin, nutshells) that mainly contain C, H, and O. Owing to their abundant carbon content, these biomass resources can be readily converted into carbon materials, which possess numerous merits as support materials for metal single atom dopants, including easy accessibility, designable surface area and porosity, rich defects and high conductivity [22, 23]. Moreover, biomass‐derived carbon materials can be tailored into versatile physical dimensions such as 0D quantum dots, 1D carbon nanotubes or nanofibers, 2D graphene, and 3D carbon sponges [24]. These characteristics provide a robust and adaptable platform for the design of Sus‐SACs. However, the scarcity of intrinsic coordination sites in carbon‐dominated precursors typically necessitates the incorporation of heteroatom sources to effectively stabilize atomically dispersed metal centers.

**FIGURE 2:**
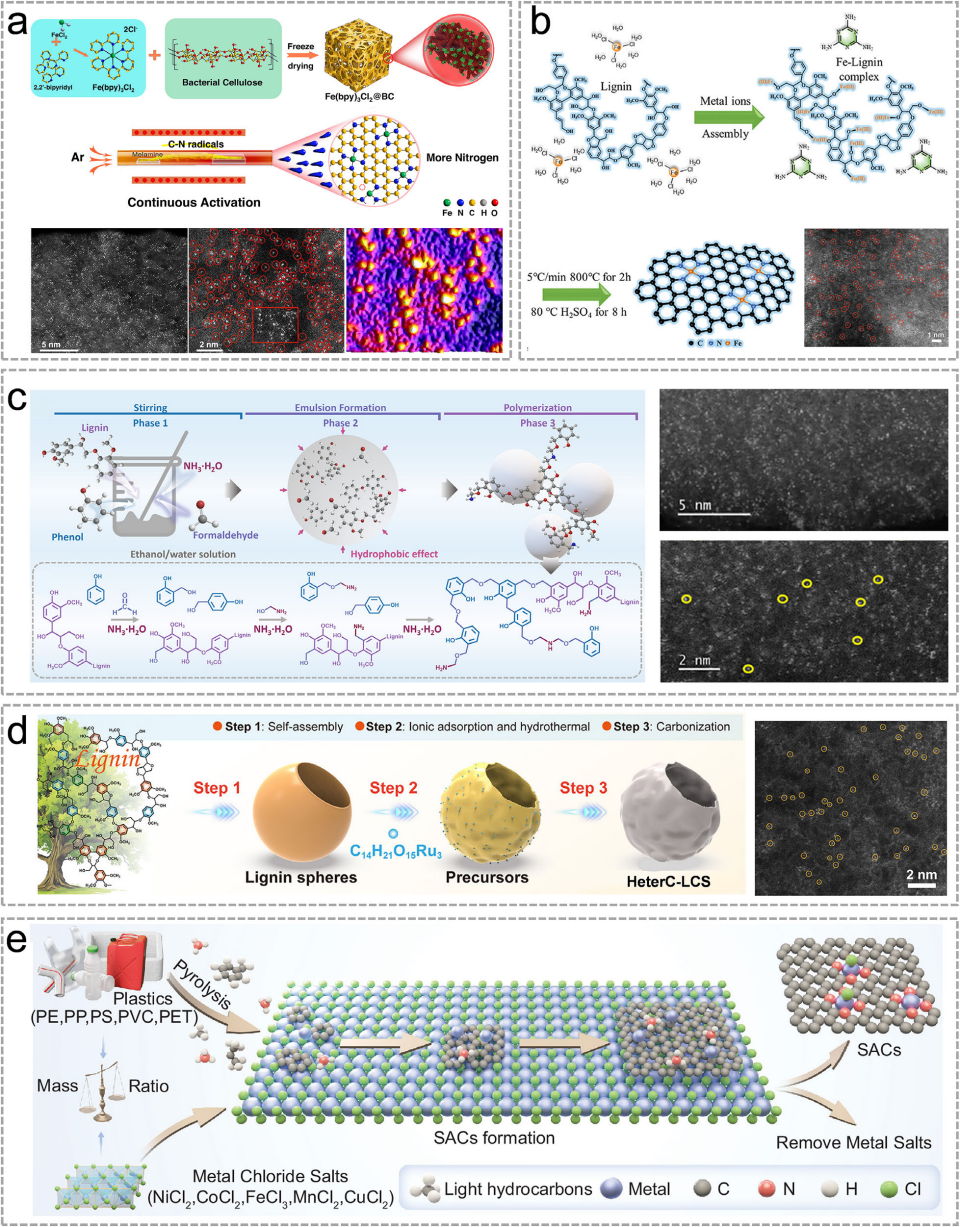
(a), Schematic illustration of the preparation process of cellulose‐derived Fe‐Sus SACs and the corresponding HADDF‐HRTEM images. Reproduced with permission [27]. Copyright 2025, The authors, Published by Springer Nature. (b), The synthesis diagram of lignin‐derived Sus‐SACs and its corresponding HADDF‐HRTEM image. Reproduced with permission [33]. Copyright 2025, Elsevier. (c), Schematic illustration of a non‐carbonization strategy for the synthesis of Pd‐Sus‐SACs using lignin‐derived resin as support. Reproduced with permission [35]. Copyright 2025, WILEY‐VCH. (d), Synthesis and morphological structure of Ru‐Sus‐SACs confined by a lignin‐derived hollow spherical nanoreactor. Reproduced with permission [36]. Copyright 2025, WILEY‐VCH. (e), Schematic illustration of Sus‐SACs derived from plastics and transition metal salts. Reproduced with permission [40]. Copyright 2025, The authors, Published by Springer Nature.

Cellulose, a linear polysaccharide composed of 1,4 β‐glycosidic bonds with ∼ 44 wt.% carbon content, is an abundant, renewable, and low‐cost biomass precursor for the synthesis of Sus‐SACs [25]. Besides, its molecular structure is rich in hydroxyl groups, providing abundant reactive sites for chemical modification [26]. In a recent study, Wang et al. developed a Fe‐based Sus‐SACs using bacterial cellulose as a carbon precursor [27]. As illustrated in Figure 2a, Fe precursor and cellulose were first freeze‐dried to form a Fe loaded porous network. Afterward, this precursor was pyrolyzed via a continuous activation (CA) strategy where a C‐N based small molecule precursor (melamine) was placed at the upstream of the Fe‐loaded SACs precursor. The continuous decomposition of melamine etched the surface oxygen groups away, inducing more vacancies for the anchoring of single atoms and reaching a high Fe atom areal density of 4.5 atoms nm^−2^ (HADDF‐HRTEM images in Figure 2a). This work demonstrated that cellulose is a superior candidate for synthesizing high areal density Sus‐SACs. Cellulose can be fabricated into porous carbon support for Fe single atoms, as verified by research from Liu et al. [28]. They also used a freeze drying‐pyrolysis strategy and the Sus‐SACs exhibited a large surface area of 1235 m^2^ g^−1^. Notably, cellulose constitutes the major component of numerous solid wastes, such as waste paper, and its utilization for the fabrication of Sus‐SACs exemplifies the concept of “turning waste into treasure.” Wu et al. successfully synthesized Co Sus‐SACs from waste paper by carbonization and acid‐washing methods [29]. By the N‐doping of polyethyleneimine, the Co SAs are existed as Co‐N_3_ configuration.

Lignin is an aromatic biopolymer that exists widely in the cell walls of vascular plants. It is the second most abundant natural polymer on Earth after cellulose, accounting for 15%–30% of plant biomass [30]. Lignin contains up to 65 wt.% of carbon, and the aromatic structure of carbon is conducive for carbonization, making it an ideal candidate for carbon materials synthesis [15]. Resembling cellulose, its abundant functional groups, such as phenolic hydroxyl groups and aliphatic hydroxyl groups, are active sites for metal ion coordination [31, 32]. Recently, Jiang et al. prepared a lignin‐based Fe Sus‐SACs via carbonization [33]. Fe metal ions were first assembled with lignin via strong and stable metal‐ligand interactions between lignin and Fe precursors as depicted in Figure 2b. Apart from Fe, Lignin also exhibited efficient interaction with Ni metal ions as demonstrated by Si et al. [34]. They used enzymatic hydrolysis lignin (EHL) from cornstalk residue as the precursor, which contains substantial phenolic hydroxyl group of 1.99 mmol g^−1^. Moreover, they introduced Zn metal atoms to replace a certain proportion of Ni sites, expanding the spatial distance between adjacent Ni atoms. This substitution ensured the desired metal loading for Ni and effectively prevented Ni aggregation. In previous researches, biomass‐derived supports for Sus‐SACs were typically obtained via high‐temperature carbonization, which often destroyed functional groups essential for strong metal–support interactions, leading to the agglomeration of metal sites. The Si group proposed a novel carbonization‐free strategy to synthesize Pd‐based Sus‐SACs by constructing nitrogen‐functionalized lignin‐based phenolic resin (N‐LPR) supports [35]. nitrogen‐functionalized lignin‐based phenolic resin (N‐LPR) supports via lignin fractionation and ammonia‐assisted polymerization. As depicted in Figure 2c, N‐LPR was obtained through lignin fractionation and ammonia‐assisted polymerization. The nano‐chain‐like N‐LPR structure has a high surface area and abundant ‐NH_2_ groups, enabling the stable anchoring of atomically dispersed Pd at room temperature. Wang et al. designed a lignin‐derived carbon–based nanoreactor to confine Ru SACs, as illustrated in Figure 2d [36]. The hollow spherical structure of this lignin‐derived nanoreactor leverages both the tip effect and the nano‐curvature effect, thereby addressing the challenges of limited active sites and sluggish catalytic kinetics.

Starch is a naturally abundant, renewable polysaccharide composed primarily of two glucose‐based macromolecules: amylose, a mostly linear chain, and amylopectin, a highly branched structure. Due to its excellent gel‐forming ability, starch is normally used to form hydrogel first and then is carbonized to obtain lightweight, porous aerogels with high surface areas and tailored pore structures [37]. In 2019, Zhang et al. employed lotus root‐derived starch and melamine as precursors to synthesize an Fe‐N coordinated hydrogel, which was subsequently subjected to a two‐step pyrolysis process to produce an N‐doped carbon aerogel with a 1.3 wt.% loading of Fe single atoms [38]. Another example is reported by Wu et al. where they prepared metal SAs decorated on N and B co‐doped porous carbon derived from soluble starch via a pyrolysis‐etching‐activation (PEA) strategy [39]. PEA strategy can be used in the universal preparation of different metal decorated Sus‐SACs, such as Co, Ni, and Fe.

Plastics constitute a substantial portion of municipal solid waste, and their extensive use, combined with inherent resistance to biodegradation, has amplified global environmental pressures. Common commercial polymers, including polyethylene (PE), polystyrene (PS), polypropylene (PP), polyethylene terephthalate (PET), and polyvinyl chloride (PVC), contain high carbon contents of roughly 85.7%, 85.7%, 92.3%, 62.5%, and 38.7% by mass, respectively. This carbon‐rich feature makes them attractive feedstocks for converting into functional carbon‐based materials. Ren et al. proposed a universal strategy to achieve library synthesisof Sus‐SACs from PE, PP, PS, PET, PVC, and mixed plastics (MPs) [40]. As depicted in Figure 2e, metal chlorides were selected as metal sources and structure templates to create porous carbon substrates. Notably, the product yield of Cu SACs was markedly higher than that of other transition metals, with CuSA‐PE exhibiting the highest yield at 88% and a batch yield of 1 gram.

### 2.2 Heteroatom‐Rich Precursors

Heteroatom‐rich precursors refer to biomass or solid waste feedstocks that contains a high proportion of elements other than carbon and hydrogen. Typical heteroatoms include nitrogen (N), sulfur (S), phosphorus (P), and boron (B) [41]. Unlike carbon‐dominated precursors, heteroatom‐rich precursors can in situ incorporate heteroatoms (e.g., N, S, and P) during pyrolysis, eliminating the need for additional external dopant sources [42]. These heteroatoms not only modulate the electronic structure and surface chemistry of the resulting carbon materials, but also act as coordination sites for stabilizing single metal atoms [17, 43]. However, the presence of abundant heteroatoms may compromise the controllability of the carbon framework and pore architecture, making precise structural regulation more challenging.

**FIGURE 3:**
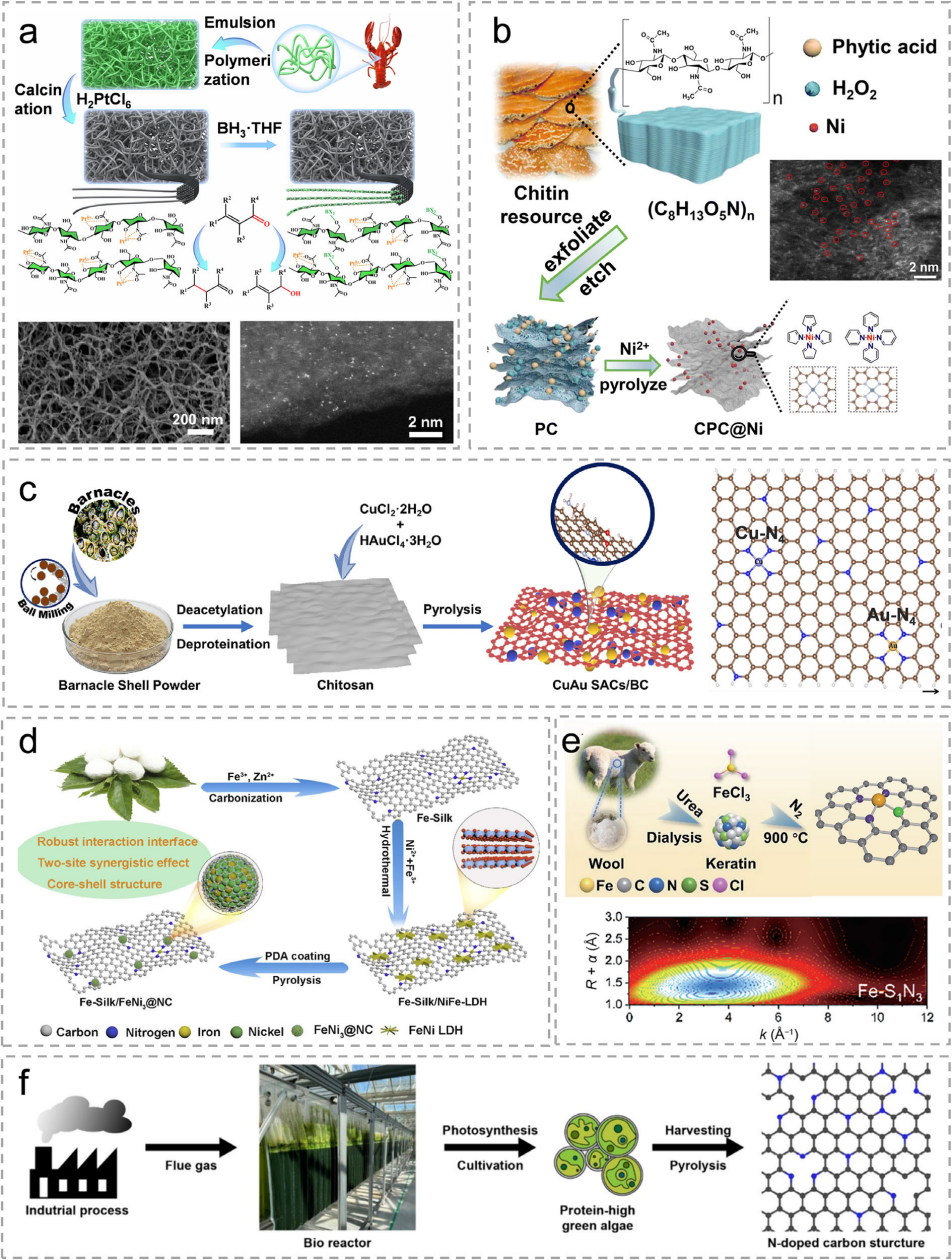
(a), The synthesis schematic illustration of supramolecular nanowires‐stabilized Pt Sus‐SACs derived from Chitin. Reproduced with permission [46]. Copyright 2025, The authors, Springer Nature. (b), The schematic diagram of the synthesis of Ni Sus‐SACs from wasted seafood. Reproduced with permission [47]. Copyright 2025, RSC. (c), The illustration for the synthesis process of Au/Cu dual metal Sus‐SACs from barnacles. Reproduced with permission [49]. Copyright 2023, Elsevier. (d), Synthetic route for the Fe Sus‐SACs from silk fibroin. Reproduced with permission [53]. Copyright 2025, Elsevier. (e), The schematic illustration of the synthesis of Fe Sus‐SACs anchored on N, S‐ doped carbon support derived from wool keratin. Reproduced with permission [55]. Copyright 2023, Springer Nature. (f), Schematic illustration of the cultivation of green algae and the preparation of algae‐derived N‐doped carbon. Reproduced with permission [57]. Copyright 2024, Elsevier.

Chitin is a polysaccharide composed mainly of N‐acetyl‐D‐glucosamine units, and it is the primary structural component in the exoskeletons of crustaceans, insects, and in the cell walls of fungi [44]. Chitin contains around 6.5‐7 wt.% N atoms, which will provide more anchor sites for metal atoms upon pyrolysis. Gan et al. synthesized Fe SACs using chitin‐derived carbon as substrate via ball milling and pyrolysis [45]. They found that the increasing pyrolysis temperature induces the formation of Fe‐N_x_ species and graphitic N. Lei et al. synthesized chitin supramolecular nanowires‐stabilized single‐atom Pt catalysts via a strong metal–organic polymer coordinative effect, as shown in Figure 3a [46]. The supramolecular nanowires were prepared by dissolving shrimp powder in a NaOH/urea solution, followed by emulsion polymerization. After freeze‐drying, the resulting nanowires were impregnated with H_2_PtCl_6_ solution and thermally activated under an Ar atmosphere to obtain the final products. The chitin nanowires provided a substrate with a high surface area (SSA) of 210 m^2^ g^−1^. The Pt loading is determined to be 1.14 wt.%. Zhu et al. extracted chitin from wasted seafood as a precursor to prepare porous chitin (PC) via an exfoliating‐etching strategy [47]. PC was further used as a porous support for the anchoring of 1.2 wt.% Ni atoms by its abundant micropores and in situ doped N elements. The synthesis strategy is shown in Figure 3b. The coordination of Ni SA sites is mainly Ni‐N_4_.

Chitosan is a polysaccharide obtained from chitin through an alkaline deacetylation process [48]. Compared with chitin, chitosan contains a larger number of free‐NH_2_ groups. Thus, chitosan contains a relatively high N content, typically 7–9 wt.%. It also possesses good solubility and high chemical reactivity in acidic solutions. In 2023, Chellasamy et al. extracted chitosan from ball milled barnacle powder via deacetylation and deproteination. Further, they fabricated gold and copper dual metal Sus‐SACs via pyrolysis as shown in Figure 3c [49]. The Cu and Au metals are anchored by N atoms within the chitosan‐derived support with a coordination structure of M‐N_4_ (M: Au/Cu) and a distance of 0.35 nm. The metal loading of Au and Cu are characterized to be 1 wt.% and 14 wt.% respectively. Yin et al. used chitosan as a starting precursor, and by controlling pyrolysis temperature, Co SACs with Co‐N_3_ and Co‐N_4_ sites were obtained [50]. Higher pyrolysis temperature leads to the formation of Co‐N_3_ coordination.

Proteins represent one of the fundamental classes of biomacromolecules and serve as essential nutrients in organisms. Structurally, proteins are polymers of amino acids, in which individual residues are covalently linked through dehydration‐condensation reactions to form peptide bonds. Owing to the structural diversity of amino acids, protein‐derived biomass is inherently enriched with various heteroatoms, offering a wide range of potential metal coordination environments. Nitrogen is the principal heteroatom, and it is incorporated through the amino groups of all amino acid residues, accounting for approximately 16% of protein mass [24]. Sulfur presents in specific amino acids such as cysteine and methionine residues, enabling the formation of disulfide bridges [51]. In 2018, Zhu et al. developed a universal strategy to synthesize SACs with M‐N sites (M  =  Fe, Co, Ni) supported by ultrathin 2D porous carbon derived from silk cocoon [52]. The silk cocoon‐derived carbon has ultra‐high SSA of 2105 m^2^ g^−1^ and a high N doping content of 9.2 wt.%. Co‐N_4_ sites are the most stable compound among M‐N_x_ structures as confirmed by density functional theory (DFT) calculations and characterization by XAS. Wang et al. reported an in situ reduction strategy for the synthesize of Fe SACs using silk fibroin‐derived carbon as support [53]. The synthesis process is illustrated in Figure 3d. The N‐doped carbon matrix from silk fibroin not only provides robust anchoring sites for Fe atoms but also forms a core–shell structure that protects metal sites from deactivation in harsh conditions. Keratin is a sulfur‐rich, fibrous structural protein that constitutes the primary component of hair, wool, nails, feathers, horns, and the outermost layers of skin [54]. In light of this, Sun et al. use wool keratin as a precursor and fabricated Fe SACs with Fe‐S_1_N_3_ sites as shown in Figure 3e [55]. By pyrolysis, wool keratin is converted into an ultrathin 2D N, S‐codoped carbon matrix. They also synthesized Fe SACs using silk fibroin‐derived carbon as support, in which the coordination structure of Fe is Fe‐N_4_. Compared with Fe‐N_4_ sites in silk fibroin‐derived SACs, keratin‐derived SACs have an optimized electronic structure for producing hydroxyl radicals.

Other biomass, such as lignin alkali and algae were also used as N containing precursor for the synthesis of Sus‐SACs. Qin et al. prepared Zinc SACs using lignin alkli as the precursor [56]. Zn SAs are coordinated with the N atoms within the lignin alkali in the form of Zn‐N_4_. To achieve atomic dispersion of Zn, the pyrolysis temperature was set at 1000°C which is beyond the evaporation temperature of the Zn atoms (908°C). Meanwhile, Zn precursor acted as the soft template during pyrolysis and its evaporation constructs a porous structure within the lignin alkali‐derived carbon support. A recent study by Lee et al. provides a representative example of synthesizing ruthenium Sus‐SACs from nitrogen‐rich algae, as shown in Figure 3f [57]. In this work, green algae (Chlorella sp. HS2) were cultivated in a bioreactor, using exhaust gas from liquefied natural gas combustion as the CO_2_ source. To achieve the maximum N‐doping, algae were harvested when reaching the size of 3–5 µm, and the N content in the obtained algae‐derived carbon is 7 wt.%. DFT calculation results demonstrated that Ru‐N_3_ are the most stable coordination structure for Ru.

### 2.3 Metal‐Rich Precursors

**FIGURE 4:**
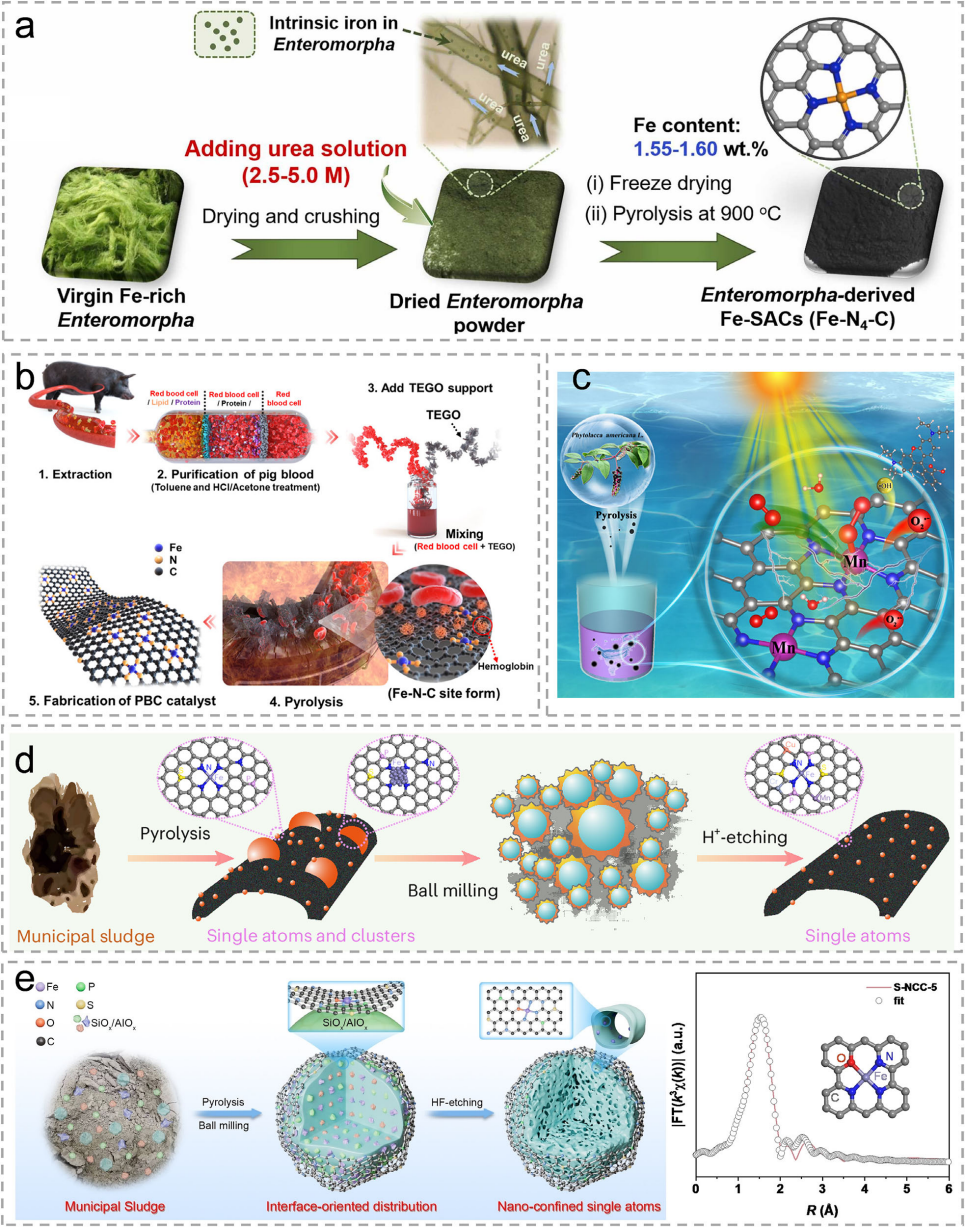
(a), Schematic illustration for the synthesis process of Enteromorpha‐derived Sus‐SACs. Reproduced with permission [58]. Copyright 2023, Elsevier. (b), Processing flow for the synthesis of pig blood‐derived Fe SACs. Reproduced with permission [61]. Copyright 2021, ACS. (c), Illustration of a Mn Sus‐SACs derived from Mn Hyperaccumulator, Phytolacca Americana. Reproduced with permission [63]. Copyright 2022, ACS. (d), Preparation diagram of sludge‐derived Fe Sus‐SACs. Reproduced with permission [65]. Copyright 2024, the authors, published by Springer Nature. (e), Illustration of the preparation process for the nanoconfined Fe Sus‐SACs from municipal waste water sludge. Reproduced with permission [66]. Copyright 2025, Wiley‐VCH.

Certain biomass resources inherently possess significant metal content, enabling their direct conversion into single‐atom catalytic sites. Algae are a typical biomass that contains rich Fe elements and can be used as a precursor for the synthesis of Sus‐SACs. Yin et al. selected Enteromorpha, an Fe‐rich marine algae, as the precursor to synthesize Fe SACs with Fe amount over 1.5 wt.% [58]. With such high Fe content, direct pyrolysis of Enteromorpha will trigger the aggregation of Fe atoms due to the lack of sufficient coordination elements such as nitrogen. Interestingly, Enteromorpha has abundant capillary structures and can adsorb nitrogen‐containing small molecules from nitrogen‐rich solutions. In light of this, the researchers enriched the N content by saturating the Enteromorpha in urea solution. The N content increased to 32.13 wt.%, which is adequate to coordinate with Fe throughout the freeze‐drying and pyrolysis processes. Their synthetic strategy is exhibited in Figure 4a. Spirulina, a widely distributed aquatic algae, contains a high protein content (up to 60%) and abundant hydrogenase with an S‐coordinated Fe center, making it a promising Sus‐SACs precursor. Lei et al. prepared a Fe SACs integrated with ultra‐small Fe_2_O_3_ nanoclusters embedded in N, S‐co‐doped porous carbon using Spirulina as N, S, and Fe source [59]. The Spirulina‐derived carbon support contains abundant edge defects. Auricularia auricular‐judae (AAJ) is a type of biomass which contains rich N and Fe. Wang et al. synthesized Fe SACs using AAJ as precursor, and Fe‐N_x_ sites are formed in situ during pyrolysis [60].

Apart from the aforementioned plant biomass, animal biomass, such as animal blood, possesses a large amount of Fe‐porphyrin in hemoglobin, which is a potential precursor for Sus‐SACs synthesis. Lee et al. used waste pig blood to synthesize Fe SACs [61]. The wasted pig blood was pretreated by toluene to remove the phospholipid layer of the blood, which is prone to coagulation. Moreover, the Fe content was further enriched by removing other protein components, except hemoglobin‐rich red blood cells, using a mixed solution of HCl and acetone. To achieve the maximum accessible active sites, they selected thermally exfoliated graphene oxide with high porosity as a 2D carbon support. The processing flow is displayed in Figure 4b.

Vascular plants are capable of absorbing metal ions such as Fe^2+^、Zn^2+^、Mn^2+^, and Cu^2^^+^from the soil and have been extensively investigated for their potential in the remediation of heavy metal‐contaminated environments [62]. Therefore, biomass harvested from hyperaccumulator plants holds potential to be used as a metal‐rich precursor for Sus‐SACs synthesis. Phytolacca americana, has been demonstrated to have a high potential to accumulate high concentrations of Mn. Cui et al. synthesized Mn SACs by carbonizing the root of Mn hyperaccumulator, P. americana, as shown in Figure 4c [63]. Mn content reaches 1.13 wt.%, and Mn‐N_4_ sites are created after pyrolysis. Li et al. fabricated Fe SACs from Fe‐enriched fern harvested from iron mines [64]. The fern derived‐ biomass waste offers a porous carbon matrix and sufficient N for Fe dispersion and coordination.

Various solid wastes are inherently rich in metal components, offering a sustainable reservoir of precursors for the synthesis of Sus‐SACs. Among them, sludge represents a typical example. Globally, municipal wastewater treatment plants generate approximately 80–90 million tons of dry sludge annually, which poses significant economic and environmental challenges. However, dry matter in sludge normally contains carbon and low‐concentration transition metals, which can be upcycled. In 2024, Yu's group developed a three‐step treatment pathway for the efficient upcycling of sludge to synthesize Sus‐SACs [65]. Their strategy is shown in Figure 4d. Municipal sludge was first pyrolyzed under 700°C, which forms a mesoporous and amorphous carbon substratewhere Fe is dispersed as SAs and clusters. Complete atomic dispersion was subsequently achieved through ball milling followed by acid leaching. They also demonstrated the large‐scale production via their strategy, yielding 540 kg of SACs from 1000 kg of dry sludge. Moreover, this strategy is universal, which is demonstrated by synthesizing Sus‐SACs from 22 sludge samples across China. Although elemental compositions of these raw sludge samples vary with operational scale, treatment processes, wastewater matrices, dewatering methods, and chemical additives, the obtained SACs nevertheless exhibit inherent compositional commonalities. In their recent work, silicon and aluminum components in the sludge are utilized to construct a mesoporous matrix which confines the Fe SA sites within its cavity [66]. The Fe species were presented within the materials with Fe‐N_3_O coordination as shown in Figure 4e. Pan et al. reported a Cu SACs synthesized from a wasted biochar sorbent saturated with Cu [67]. The biochar adsorbent has inherent advantages as a matrix, for it has a very high specific surface area and highly developed pore channels, as well as rich Cu content as a metal source. Cu atoms are anchored by the N atoms within the biochar adsorbent.

Overall, Metal‐rich precursors serve as self‐sacrificial metal sources that promote the in situ generation of atomically dispersed sites while reducing reliance on external metal salts and thus simplifies the synthetic procedure. Nevertheless, the metal loading is typically low and often difficult to precisely control.

## 3 Synthesis Methods of Sus‐SACs

**FIGURE 5:**
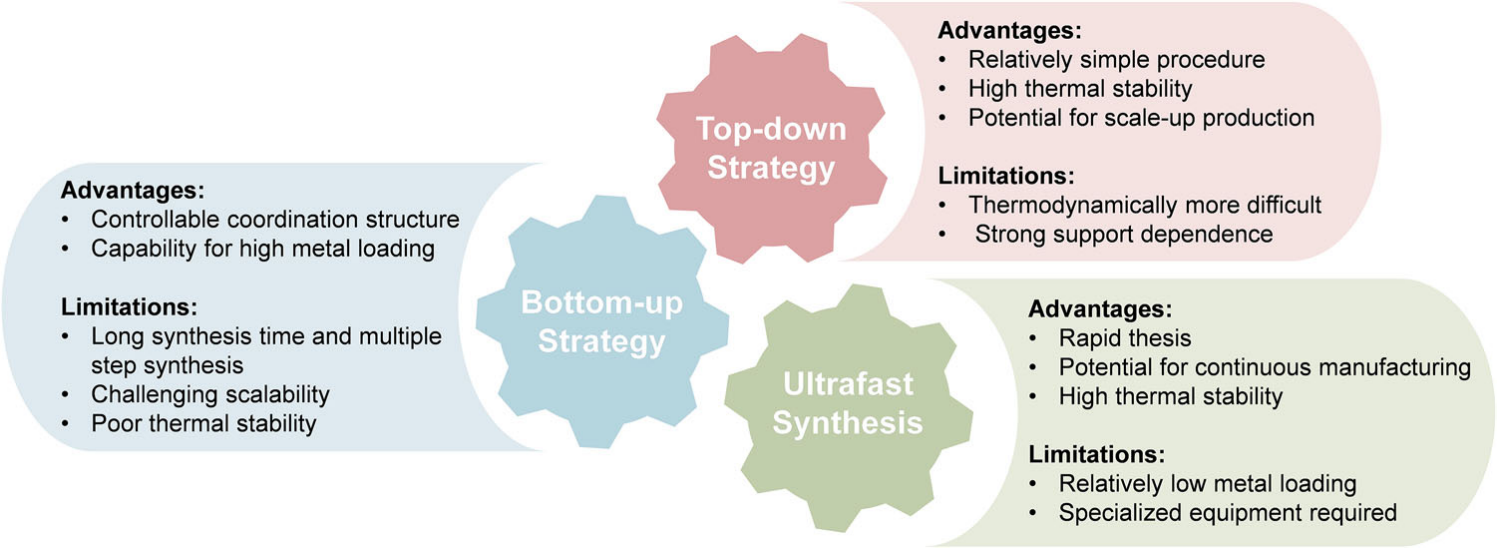
Schematic illustration summarizing the advantages and limitations of the major synthetic strategies for Sus‐SACs [72, 73, 74].

Apart from precursor selection, the synthesis strategy plays a crucial role in determining the final structure and performance of Sus‐SACs. In many cases, precursors undergo modification pretreatment to obtain structures more suitable for anchoring metal single atoms, such as porous frameworks or heteroatom‐rich matrices. Common pretreatment approaches include hydrothermal treatment, activation, and freeze‐drying, etc. An effective synthetic method should achieve the atomic dispersion of metal atoms while prevent metal aggregation, which remains both crucial and challenging. At present, common synthesis approaches can be broadly classified into two categories: bottom‐up and top‐down [68]. In the bottom‐up strategy, metal atoms are atomically dispersed within the precursor and subsequently confined by the support through various reduction methods [69]. By contrast, the top‐down strategy seeks to isolate individual metal atoms from nanoparticles or even larger‐scale metal precursors [70]. Traditional synthesis methods, such as pyrolysis, atomic layer deposition (ALD), and co‐precipitation, often suffer from low yield, complex processing procedures, and long processing times, which hinders their large‐scale application. Recently, the emergence of ultrafast synthesis methods, for example, joule heating, laser ablation and microwave heating, with features of rapid heating and instantaneous quenching, has provided a promising route for the scalable synthesis of Sus‐SACs [71]. This section presents representative pretreatment approaches, conventional synthetic routes, and emerging ultrafast strategies for the preparation of Sus‐SACs. To enable a more systematic comparison, Figure 5 summarizes the key advantages and inherent limitations of the major synthesis categories.

### 3.1 Support Modification and Pretreatment

**FIGURE 6:**
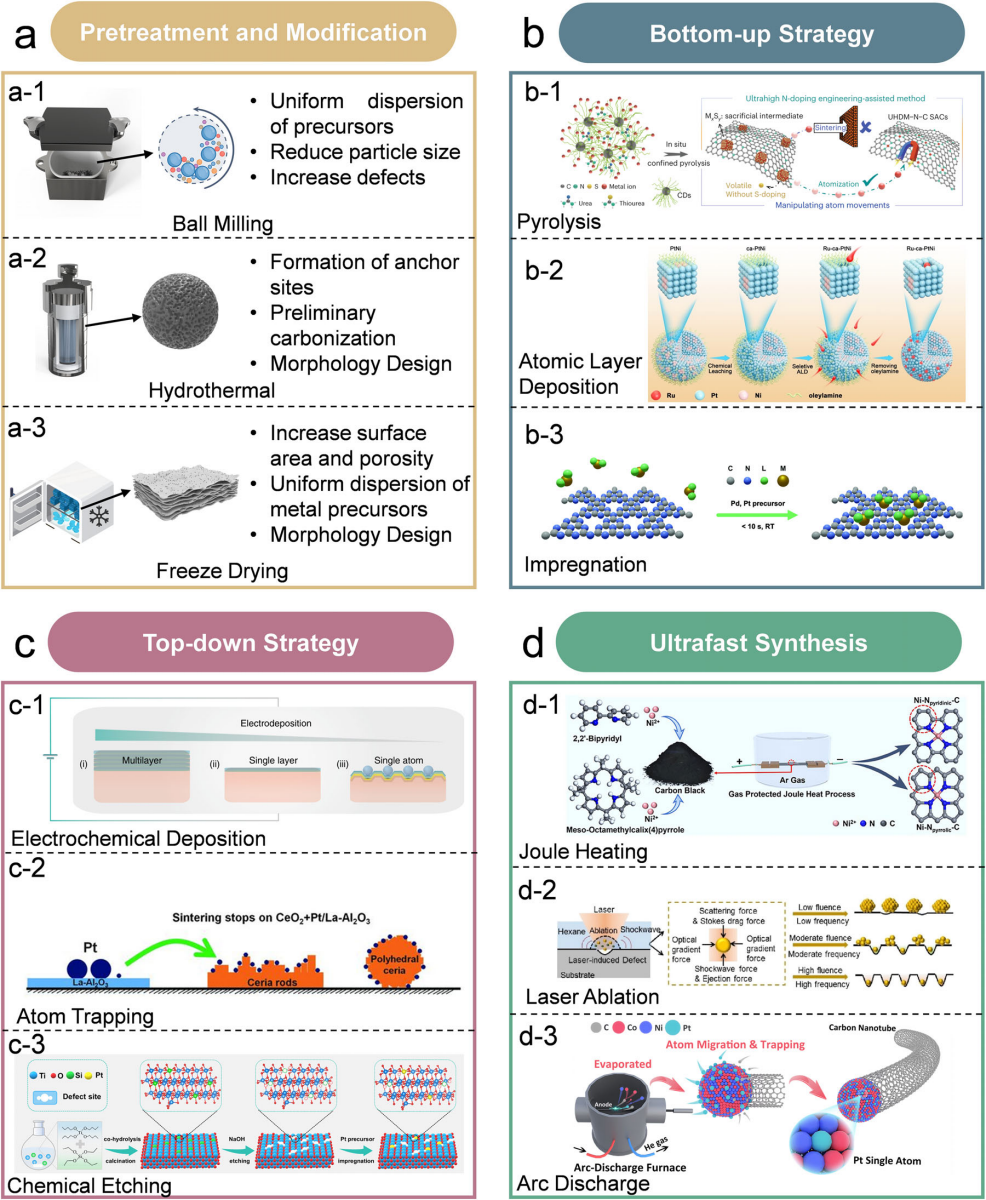
(a), Pretreatment and modification approaches for Sus‐SACs synthesis: (a‐1), Ball milling method. (a‐2), Hydrothermal method. (a‐3), Freeze drying. (b), Bottom‐up synthesis approaches of SACs: (b‐1), Pyrolysis. Reproduced with permission [118]. Copyright 2024, Springer Nature. (b‐2), Atomic layer deposition. Reproduced with permission [119]. Copyright 2022, Wiley‐VCH. (b‐3), Impregnation. Reproduced with permission [120]. Copyright 2023, the authors, published by Wiley‐VCH. (c), Top‐down synthesis approaches of SACs: (c‐1), Electrochemical deposition. Reproduced with permission [121]. Copyright 2020, the authors, published by Springer Nature. (c‐2), Atom trapping. Reproduced with permission [95]. Copyright 2016, the American Association for the Advancement of Science. (c‐3), Chemical etching. Reproduced with permission [98]. Copyright 2025, the American Chemical Society. (d), Ultrafast synthesis of SACs: (d‐1), Joule heating. Reproduced with permission [103]. Copyright 2025, Wiley‐VCH. (d‐2), Laser ablation. Reproduced with permission [106]. Copyright 2023, the American Chemical Society. (d‐3), Arc discharge. Reproduced with permission [110]. Copyright 2025, the authors, published by Wiley‐VCH.

The mixing of metal precursors with biomass or solid‐waste precursors is crucial for achieving uniform metal dispersion. Manual grinding is used for the initial and simple mixing of precursors. By contrast, ball milling is a stronger mechanical process which is effective in reducing particle size and mixing multiple solids uniformly (Figure 6a‐1) [75, 76]. Zhang et al. studied the effect of ball milling time on the structure of soybean protein‐derived carbon [77]. Prolonged ball milling time can increase the SSA of carbon support, increasing from 935 m^2^ g^−1^ for 10 h ball milling to 1211 m^2^ g^−1^ for 18 h ball milling. Moreover, ball milling is also an effective approach to create defects in carbon support [75]. Zafir et al. used ball milling and carbonization to produce N‐doped graphene powder from palm kernel shell (PKS) [78]. Ball milling of the carbonized PKS at 500°C for 90 min produced graphene powder with an I_D_/I_G_ ratio of 0.65 of 0.33. The significantly higher I_D_/I_G_ value compared to 0.19 for commercial graphene indicates the presence of structural defects within the material.

Hydrothermal method is widely adopted to modify the structure of Sus‐SACs support (Figure 6a‐2). The principle of hydrothermal carbonization (HTC) is to replicate the natural coalification process within a short time through the combined effects of elevated temperature and pressure. During the hydrothermal process, Sus‐SACs precursors undergo several reactions, such as condensation, polymerization, hydrolysis, decarboxylation, dehydration, and aromatization, inducing the preliminary carbonization of the carbon source and the formation of functional group‐rich structures [79]. These surface functional groups can serve as coordination or anchoring sites for metal ions in subsequent steps for the formation of single atoms. Moreover, the hydrothermal method enables the formation of tailored morphologies. For example, Zhu et al. utilized this approach to exfoliate the hydrogen bonds between chitin molecular chain layers, resulting in a coral cluster‐like carbon matrix suitable for Sus‐SACs synthesis [47].

Freeze‐drying is a dehydration technique that removes moisture by sublimating ice under low temperature and vacuum (Figure 6a‐3). During the freeze‐drying process, the precursor solution can be uniformly coated onto or embedded within the gel network. This collapse‐free drying approach yields carbon aerogels with extremely low density, high porosity, and a large specific surface area, providing ample active sites and dispersion space for subsequent single‐atom anchoring. Guo et al. adopted freeze‐drying method for the modification of cellulose‐derived precursor and obtained a matrix with a SSA of 1235 m^2^ g^−1^ [28]. Guo et al. obtained a fibrous carbon matrix from bacterial cellulose via freeze‐drying, and the SSA of the matrix reaches 602 m^2^ g^−1^ [27].

### 3.2 Bottom‐Up Synthesis of Sus‐SACs

Pyrolysis represents one of the most widely adopted bottom‐up strategies for the synthesis of Sus‐SACs (Figure 6b‐1). In this approach, metal precursors and biomass‐ or solid‐waste‐derived carbon sources are homogeneously mixed and then subjected to high‐temperature thermal decomposition under an inert atmosphere. Pyrolysis temperature plays a key role in determining the final structure of Sus‐SACs. Increasing the pyrolysis temperature generally enhances the graphitization degree of the carbon support, while simultaneously reducing the heteroatom content [80]. Elevating the temperature is the most straightforward way to achieve atomization. However, under high temperatures, isolated metal atoms on supports often undergo migration and aggregation, leading to the formation of clusters or larger nanoparticles. The trade‐off effect of atomization and sintering at high pyrolysis temperature makes it challenging to synthesize high‐metal loading SACs from pyrolysis. Recently, several strategies have emerged for the synthesis of high metal loading SACs via pyrolysis [81, 82]. Wang et al. developed a negative pressure annealing strategy which achieves the synthesis of SACs with metal contents up to 27.3–44.8 wt.% for 13 different metals on carbon nitride matrix [83]. By applying negative pressure during annealing, anionic ligands are removed more rapidly from metal precursors. This process enhances the bonding between the metal species and nitrogen‐deficient sites, leading to the creation of a high density of metal sites coordinated with nitrogen. Using a metal sulfide‐mediated atomic trapping method, Chang et al. produced 17 distinct single‐atom catalysts (SACs) featuring metal loadings exceeding 20 wt.%. They also extended this approach to create high‐entropy SACs with five and twenty different metals, all possessing ultrahigh metal contents [84]. The formation of highly dispersed single metal atoms was achieved by manipulating the metal‐atom migration pathway. This was accomplished using an ultrahigh N‐doped support, synthesized from carbon dots, thiourea, and urea, which provided sufficient anchoring sites to capture the atoms.

Atomic layer deposition (ALD) is a powerful bottom‐up strategy for the synthesis of SACs (Figure 6b‐2). ALD ensures that metal atoms are deposited individually and become covalently bonded to the support's functional groups, effectively preventing aggregation [85].ALD enables the precise engineering of synergistic single‐atom and atomic cluster dual‐sites, which enhances the catalytic performance by their synergistic effects.[86] Xu et al. employed an amino group‐assisted ALD strategy and achieved controllable construction of SiO_2_‐supported Pt catalysts with Pt SAs and Pt atomic clusters [87]. The amino functionalization process of the SiO_2_ support controls the dispersion behavior of Pt, stabilizing it as single atoms and effectively preventing nanoparticle formation, which was observed on the unmodified SiO_2_. Impregnation is one of the most common methods for synthesizing SACs. The process involves immersing a support material into a precursor solution, where metal ions are anchored via electrostatic adsorption onto the surface of the support, followed by steps, such as rotary evaporation, drying, and reduction [88]. Moragues et al. designed a droplet‐based microfluidic reactors for the impregnation synthesis of Pt and Pd SACs on C_3_N_4_ support, as shown in Figure 6b‐3 [89]. Rapid mixing within nanoliter microfluidic droplets allows for precise control of support particle residence times and metal precursor concentrations.

### 3.3 Top‐Down Synthesis of Sus‐SACs

The top‐down approach starts from metal nanoparticles or bulk metal materials and transforms them into isolated atomic sites on a support through various treatment techniques. Unlike bottom‐up routes, this method does not rely on atomically dispersed precursors, which simplifies the overall synthesis and lowers production costs. Additionally, during the downsizing of bulk metals to single atoms, numerous structural defects and vacancies can be introduced, providing more active sites for catalysis [90, 91]. Electrochemical deposition offers a simple and versatile approach for synthesizing SACs, as shown in Figure 6c‐1. In this process, the substrate functions as the working electrode, while a metal source serves as the counter electrode. Electrochemical deposition methods for fabricating SACs mainly include overpotential deposition (OPD) and underpotential deposition (UPD) [92]. It is well recognized that maintaining a relatively low precursor concentration, along with proper scanning cycles and rates, helps achieve isolated single‐atom deposition by preventing excessive supersaturation [93].

It is commonly recognized that metal nanoparticles on oxide supports are prone to sintering under oxidative high‐temperature conditions. This sintering process leads to a significant loss of metal dispersion and surface area, which reduces catalytic performance [94]. In 2016, Jones et al. developed an atom trapping strategy to prevent the sintering of Pt 800°C in oxidizing ambient conditions using a CeO_2_ support, as shown in Figure 6c‐2 [95]. Moreover, the atom trapping effect shows a shape‐dependent characteristic. This study demonstrated that polyhedron‐ and rod‐like nanoceria supports were more efficient in trapping Pt single atoms compared to cubic ones. Chemical etching is a widely used technique for synthesizing SACs. The controlled etching process not only enhances the dispersion of single metal atoms but also introduces defects and vacancies, which serve as anchoring sites for the single atoms, promoting their stability [96, 97]. As shown in Figure 6c‐3, the recent work by Zou et al. used NaOH as etching reagent and created abundant defect sites on Ti‐O‐Si composite support for the effective mount of Pt SAs [98].

### 3.4 Ultrafast Synthesis of Sus‐SACs

Ultrafast synthesis methods have the features of rapid heating, ultrahigh temperature, and fast quenching. These approaches offer an efficient and convenient route for the rapid synthesis of sustainable single‐atom catalysts (Sus‐SACs) and hold strong potential for meeting the demands of large‐scale industrial production [99]. Joule heating, also referred to as ohmic heating, is an emerging rapid‐heating technique derived from Joule's law. In this process, the potential difference generated across a conductor accelerates charge carriers, which then collide with lattice ions, intensifying atomic vibrations. As a result, the electrical energy is efficiently converted into thermal energy, enabling ultrafast heating within the material [100]. The heating rate of the Joule heating method can reach 10^3–^10^5^ K s^−1^ [101, 102]. Subsequent fast quenching process can prevent Ostwald ripening and nanoparticle aggregation. The Joule heating synthesis process is remarkably fast, typically completed within just a few seconds. Beyond its advantage of ultrafast synthesis, this technique also enables the rational design of SACs. For example, Chen et al. utilized Joule heating to prepare Ni SACs and successfully obtained two types of single Ni atom electrocatalysts featuring distinct pyridinic‐N and pyrrolic‐N coordination environments, as depicted in Figure 6d‐1 [103]. The pyridinic‐N configuration induces a high‐spin state in the Ni single‐atom sites, whereas the pyrrolic‐N environment stabilizes a low‐spin state. The high‐spin Ni SA site exhibits an exceptional performance in electrocatalytic CO_2_ reduction.

The laser ablation in liquid (LAL) technique has emerged as a promising strategy for fabricating SACs. Laser pulses are utilized to generate defects on the substrate and decompose metal precursors, leading to the formation of isolated single metal atoms [104]. The laser energy was absorbed and converted to heat energy, and a high temperature within the femtoliter focal volume was generated on the target material [105]. Wang et al. proposed a “digging‐planting” mechanism of the LAL method for SACs synthesis as shown in Figure 6d‐2 [106]. During the “digging” stage, multiphoton absorption and optical resonance effects generate extremely high temperatures within a confined focal region, leading to the formation of abundant surface defects on the substrate. These defects alter the local electronic and coordination environment, which creates active sites for anchoring single atoms. The concentration and type of defects depend primarily on laser fluence and frequency, which determine the extent of lattice perturbation. In the subsequent “planting” stage, the same laser energy decomposes metal precursors into individual atoms while inducing plasma and shockwave effects that drive atomic motion. The interplay of outward and inward forces, such as ejection, scattering, and optical gradient forces, facilitates a self‐positioning process. Through this dynamic interaction, the atoms become stabilized on the substrate as isolated single atoms or small clusters, enabling the controlled formation of SACs with high dispersion and stability.

The direct current arc discharge (DCAD) method utilizes direct current at high voltage to create an electric arc between two electrodes [107]. It is similar to the existing flash thermal‐quenching procedure, but superior in providing an estimated core temperature of 4000–10000 K, [108] which is high enough to vaporize all the elements. Subsequently, the resulting atomic vapor rapidly migrates and reassembles at a quenching rate of approximately 166 K ms^−1^ on the cooling water‐jacketed chamber wall [109]. Our group recently reported a one‐pot strategy via the DCAD method for the gram‐scale synthesis of Pt SACs, as shown in Figure 6d‐3 [110]. The precursors are straightforward, consisting of metallic cobalt (Co), nickel (Ni), Pt oxide, and graphite, which are physically mixed beforehand. The Co and Ni metals are anticipated to condense into a CoNi alloy, which then serves to trap and stabilize the Pt single atoms. This forms a stable binding that remains intact even at the extremely high temperatures generated by the arc discharge system, thereby ensuring the high stability of the Pt single atoms. The obtained Pt SACs possess a high areal density of 10 atoms nm^−2^. Moreover, we further demonstrated its universality in extending to other noble metal SAC synthesis, such as iridium (Ir).

As discussed above, the choice of precursors, pretreatment strategies, and synthesis methods all critically influence the properties of the resulting Sus‐SACs. To facilitate the rational design of these catalysts, Table 1 summarizes the commonly used raw materials, heteroatom sources, synthesis methods, product yields, SA coordination environments, and the physical characteristics of the obtained Sus‐SACs.

**TABLE 1:** A Summary of raw materials, synthesis methods, SA coordination environments, and physical characteristics of reported Sus‐SACs.

Sus‐SACs	Raw Sources	Carbon Structure	Hetero‐atom doping	Synthesis Method	Single Atom	Loading Mass (wt %)	Coordination	Surface Area (m^2^ g^−1^)	Yield	Reference
CA‐Fe@BC	Bacterial Cellulose	Hollow fibers	N from melamine	Freeze Drying & Pyrolysis	Fe	2.60	Fe‐N_4_	601.6	Milligram Scale	[27]
FeNPC	Cellulose	Porous Carbon	• N from Urea • P from Na_3_PO_4_	Freeze Drying & Pyrolysis	Fe	0.89	Fe‐N_3_P	1235	Milligram Scale	[28]
Co‐N_3_/WPAC	Waste paper	Porous carbon	N from Polyethyleneimine	Pyrolysis	Co	/	Co‐N_3_	535.2	Milligram Scale	[29]
SA Fe‐N/C	Lignin from Bamboo	Porous carbon	N from melamine	Pyrolysis	Fe	2.69	Fe‐N_4_	429.8	Milligram Scale	[33]
Ni SAs‐N@LC	Enzymatic hydrolysis lignin from cornstalk residue	Wrinkled carbon sheets	N from melamine	Pyrolysis	Ni	1.2	Ni‐N_3_	/	Milligram Scale	[34]
Pd@N‐L3PR‐50%	Lignin	N‐functionalized lignin‐based phenolic resins	N from NH_3_·H_2_O	Stöber Method	Pd	∼3.3	Pd‐N_3_	218.8	Milligram Scale	[35]
NCA_LR_/Fe.	Lotus root‐derived starch	Carbon aerogel	N from melamine	Pyrolysis	Fe	1.3	Fe‐N_4_	699.8	Milligram Scale	[38]
ISAS Co/NBPC	Soluble starch	Carbon aerogel	N from dicyandiamide B from boric acid	Pyrolysis	Co	1.9	Co‐N_3_B	638.3	Gram scale	[39]
HeterC‐LCS	Lignin	Hollow spherical carbon nanoreactor	/	Hydrothermal & Pyrolysis	Ru	1	Ru‐N_4_	/	Milligram Scale	.[36]
CuSA‐PE	Plastics	Porous Carbon	N from Ammonia Gas	Pyrolysis	Cu	0.24	Cu‐N_4_	1958	Gram Scale	[40]
Fe_1_CNCl	Glucose	3D honeycomb‐like Carbon	N from Dicyandiamide Cl from NaCl salt	Pyrolysis	Fe	/	Cl_1_‐Fe‐N_4_	/	Milligram Scale	[111]
Fe_1_/NC‐700	Chitin	Bulk Carbon	In situ N	Ball milling & Pyrolysis	Fe	1.14	Fe‐N_x_	5.5	Gram Scale	[45]
SS‐Pt‐CSNs	Chitin	Carbon nanowires	In situ N	Freeze Drying & Pyrolysis	Pt	1.14	Pt‐N/O	210	Milligram Scale	[46]
CPC@Ni	Chitin	Porous Carbon	In situ N	Hydrothermal & Pyrolysis	Ni	1.4	Ni‐N_4_	1107	Milligram Scale	[47]
CuAu SACs/BC	Chitosan	N‐doped Carbon	In situ N	Pyrolysis	Au & Cu	Au:1 Cu:14	Au‐N_4_ Cu‐N_4_	/	Milligram Scale	[49]
Co‐N_3_ and Co‐N_4_ catalysts	Chitosan	N‐doped Carbon	In situ N	Pyrolysis	Co	∼2.5	Co‐N_4_ (900°C) Co‐N_3_ (1050°C)	/	Gram Scale	[50]
FeSAs‐NiCo alloy@N‐C	Chitosan	N‐doped Carbon	In situ N and Melamine	Pyrolysis	Fe	2.23 At. %	Fe‐N_x_	/	Gram Scale	[112]
Co–N–B–C	Chitosan	Porous Carbon	In situ N and Boron from Boric Acid	Pyrolysis	Co	4.2	Co‐N‐B‐C	1056	Milligram Scale	[113]
Co‐ISA/CNS	Silk Fibroin	Ultrathin 2D Porous Carbon	In situ N	Pyrolysis	Co	0.6	Co‐N_4_	2105	Gram Scale	[52]
SAFe/CNS	Silk Fibroin	Porous Carbon	In situ N	Pyrolysis	Fe	0.57	Fe‐N_4_	1970	Gram Scale	[114]
Fe‐Silk/FeNi_3_@NC	Silk Fibroin	Porous Carbon	In situ N	Hydrothermal & Pyrolysis	Fe	0.57	Fe‐N_4_	566	Milligram Scale	[115]
Fe‐S_1_N_3_ SAC	Wool Keratin	Ultrathin 2D Porous Carbon	In situ N, S	Pyrolysis	Fe	1.16	Fe‐S_1_N_3_	305	Milligram Scale	[55]
Fe–N/C‐SAC	Pork Liver	Porous Carbon	In situ N	Pyrolysis	Fe	2.6	Fe‐N_4_	803	Gram Scale	[116]
ZnN_4_‐SAC	Lignin Alkali	2D Porous Carbon	In situ N, S	Pyrolysis	Zn	0.67	Zn‐N_4_	584	Gram Scale	[56]
1Ru/GA5Mg	Green Algae	Porous Carbon	In situ N	Pyrolysis	Ru	0.56	Ru‐N_3_	582	/	[57]
Enteromorpha‐derived Fe‐SACs (Fe‐N_4_‐C)	Enteromorpha	N‐doped Carbon	In situ N	Freeze Drying & Pyrolysis	Fe (in situ)	1.5	Fe‐N_4_	36	Gram Scale	[58]
Enteromorpha‐derived Fe‐SACs (Fe‐N‐C)	Enteromorpha	N‐doped Carbon	In situ N	Pyrolysis	Fe (in situ)	0.84	Fe‐N_2_O_2_	/	Gram Scale	[117]
Fe_SA_/FeO_NC_/NSC	Spirulina	N, S‐doped Carbon	In situ N, S	Pyrolysis	Fe (in situ)	0.4	Fe‐N_4_	1927	Gram Scale	[59]
Fe‐ISA/NC	Auricularia auricular‐judae	N‐doped Carbon	In situ N	Pyrolysis	Fe (in situ)	0.1	Fe‐N_4_	1107	/	[60]
PBC/900/M	Wasted Pig Blood	2D Porous Carbon	NH_3_	Pyrolysis	Fe (in situ)	1.9	Fe‐N_4_	607	/	[61]
PABC‐750	Mn Hyperaccumulator, Phytolacca Americana	Porous Carbon	In situ N	Pyrolysis	Mn (in situ)	1.13	Mn‐N_4_	470	/	[63]
FeSAC‐800	Fern	Porous Carbon	NH_3_∙H_2_O	Pyrolysis	Fe (in situ)	/	Fe‐N_4_	276	Gram Scale	[64]
PME‐700	Sludge	Mesoporous Carbon	In situ N, S, P	Pyrolysis‐Ball Milling‐ Acid Leaching	Fe (in situ)	0.99	Fe‐N_4_	203	Ton scale	[65]
S‐NCC‐5	Sludge	Mesoporous Carbon	In situ N, S, P	Pyrolysis‐Ball Milling‐ Acid Leaching	Fe (in situ)	0.95	Fe‐N_3_O	246	Gram Scale	[66]
3SACu@NBC	Biochar Adsorbent	Porous Carbon	N from Dicyandiamide	Pyrolysis	Cu (in situ)	3.41	Cu‐N_4_	183	/	[67]

## 4 Characterization Techniques of Sus‐SACs

**FIGURE 7:**
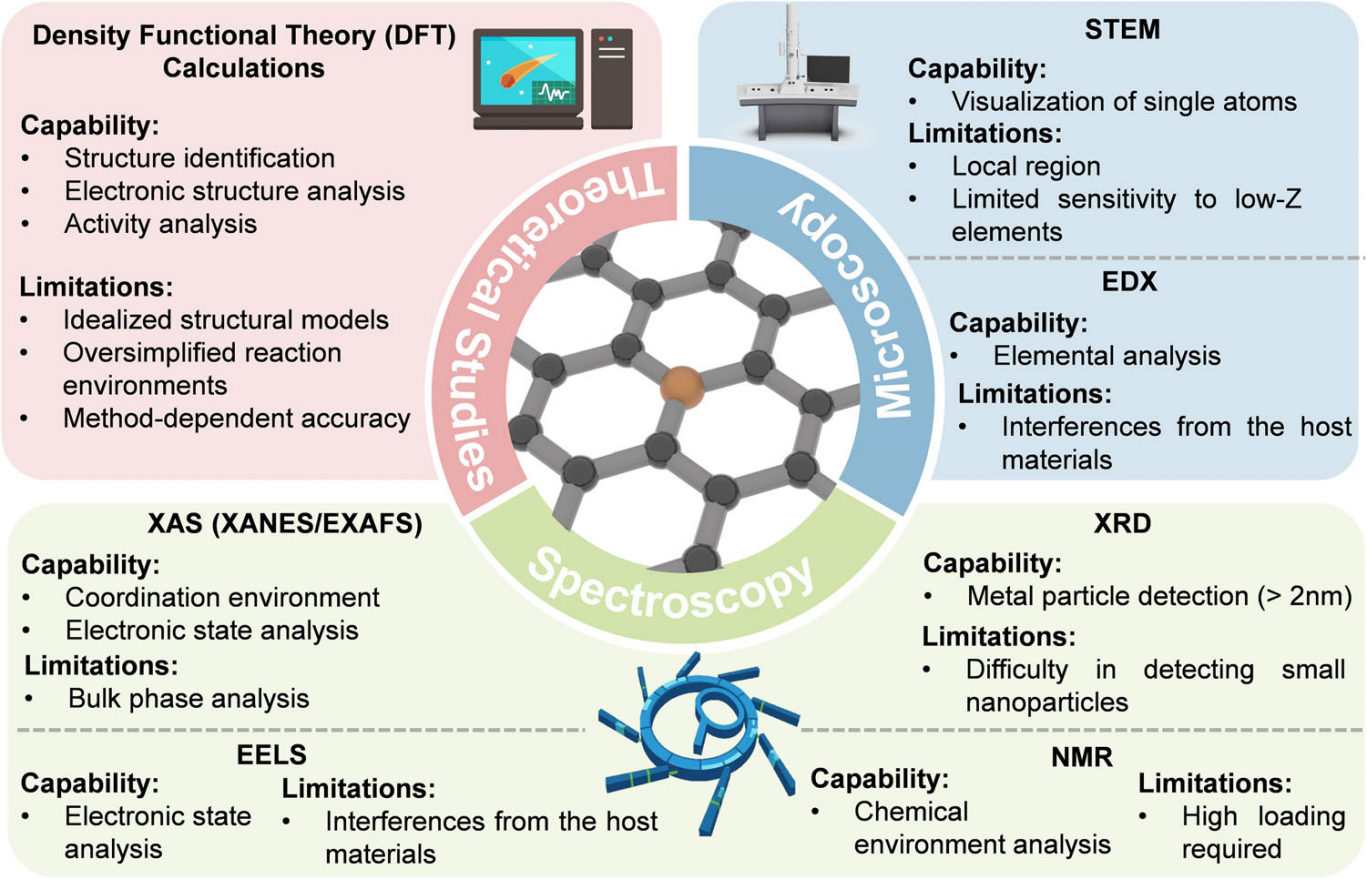
Comparative overview of the capabilities and limitations of the most widely employed characterization techniques for Sus‐SACs [123].

Determining the atomic‐level geometric, electronic structures, along with the coordination environment, is crucial for understanding the structure‐activity relationship of Sus‐SACs. This insight is fundamental for designing and optimizing active sites tailored to specific catalytic reactions [122]. The characterization of Sus‐SACs requires a comprehensive toolbox of complementary techniques, including microscopy, spectroscopy, and theoretical approaches. Figure 7 compares the capabilities and limitations of the most widely applied methods. This section provides a concise overview of their current scope and recent advances in SAC characterization.

**FIGURE 8:**
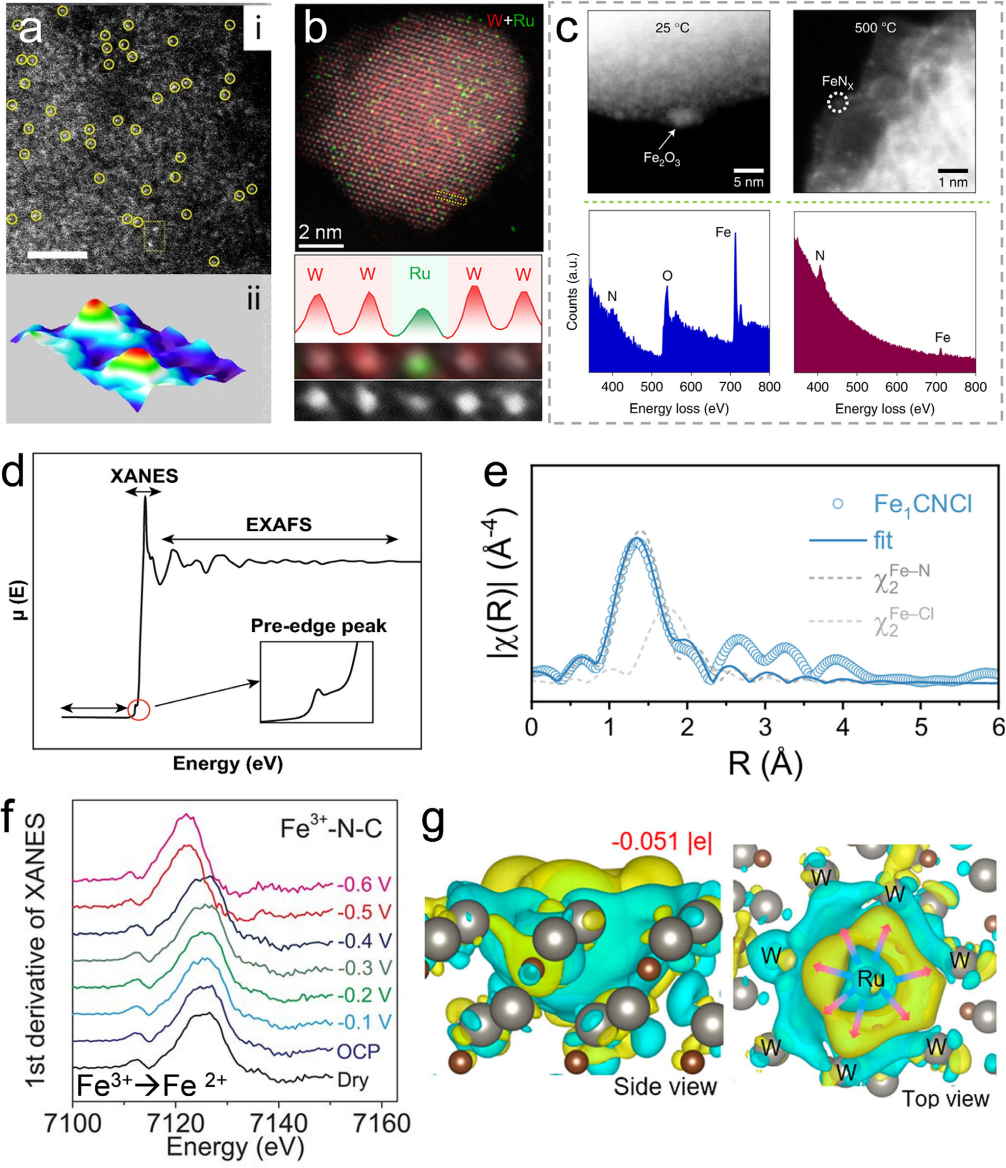
(a), AC‐HAADF–STEM image (i) and atom‐overlapping image (ii) for SACs characterization. Reproduced with permission [65]. Copyright 2024, Springer Nature. (b), Atomically resolved EDS elemental mapping of SACs. Reproduced with permission [132]. Copyright 2024, the American Chemical Society. (c), EELS spectra of Fe SACs. Reproduced with permission [133]. Copyright 2022, Springer Nature. (d), Schematic of XAS including the pre‐edge, XANES, and EXAFS regions. Reproduced with permission [134]. Copyright 2019, the Authors, Published by Springer Nature. (e), EXAFS fitting curves in R space. Reproduced with permission [111]. Copyright 2025, the Authors, Published by Springer Nature. (f), Operando EXAFS spectra in R space during electrocatalysis. Reproduced with permission [130]. Published by the American Association for the Advancement of Science. (g), Bader electron transfer by DFT calculation. Reproduced with permission [132]. Copyright 2024, the American Chemical Society.

Aberration‐corrected high‐angle annular dark‐field scanning transmission electron microscopy (AC‐HAADF‐STEM) is a powerful approach to visualize the structure of SA sites. With the incorporation of aberration correctors, the resolution of STEM can achieve the atomic level. The STEM signal follows a Z^n contrast, where it is proportional to the nth power of the atomic number [124]. Because heavy atoms scatter electrons more strongly, they appear as brighter spots in the images. The dispersion of SAs can be clearly visualized when the atomic number of the metal species is substantially higher than that of the support (Figure 8a). Given that the supports of Sus‐SACs are predominantly carbon, AC‐HAADF‐STEM provides an effective means of characterizing their single‐atom distribution. However, for supports that contain metal elements, such as metal oxides, the contrast between single atoms and the support atoms becomes much weaker, posing challenges for their direct visualization [125]. To overcome this limitation, AC‐HAADF‐STEM is often combined with energy‐dispersive X‐ray spectroscopy (EDS) and electron energy‐loss spectroscopy (EELS) [126]. EDS identifies the elemental composition of a sample by detecting characteristic X‐rays emitted upon electron‐beam excitation. Elemental analysis at the atomic scale can be achieved by integrating EDS with AC‐STEM owing to its high spatial resolution, as shown in Figure 8b. EELS can provide electronic‐structure information at the single‐atom level, including details such as the atom's oxidation state and coordination environment (Figure 8c).

X‐ray adsorption spectroscopy (XAS) based on synchrotron radiation has emerged as a crucial technique for probing the electronic structure and local coordination environment of metal SA sites [127]. XAS spectroscopy encompasses two complementary regions: the extended X‐ray absorption fine structure (EXAFS) and the X‐ray absorption near‐edge structure (XANES) as depicted in Figure 8d [128]. EXAFS analyzes analyzing the scattering signals from atoms surrounding the absorber, which enables a qualitative evaluation of the coordination environment of the target atom. Additionally, the least‐squares fitting analysis of EXAFS data allows the determination of the coordination bond lengths and coordination numbers of single atoms (Figure 8e). XANES focuses on probing the electronic structure and oxidation state of the absorbing atom. By examining the excitation of core electrons into the valence and conduction bands, it provides detailed evidence of the interactions between SA sites and their supporting substrates. In Sus‐SACs, metal sites are normally loaded on non‐metal supports. Thus, the atomic dispersion of metal atoms can be demonstrated by the absence of a metal‐metal bond in EXAFS. In situ XAS characterization has emerged as powerful and widely applied techniques for monitoring the electronic structure, atomic configuration, and size evolution of SACs under real operating conditions such as electrocatalysis [129]. For example, the reduction of Fe^3+^ to Fe^2+^ can be observed by in situ XAS under during CO_2_ electroreduction reaction, as shown in Figure 8f [130].

Many other techniques are also adopted to assist the analysis of Sus‐SACs. X‐ray diffraction (XRD) spectroscopy, particularly synchrotron XRD, is commonly employed to verify the atomic dispersion of metal sites. When X‐rays interact with a crystalline material, they are scattered by the constituent atoms in an ordered manner, producing a characteristic diffraction pattern. Analysis of this pattern enables inference of the sample's composition and underlying crystal structure. However, this technique cannot resolve metal species smaller than 1–2 nm unless they are periodically incorporated into a crystalline lattice. Consequently, it can only confirm the absence of larger metal particles [123]. Nuclear magnetic resonance (NMR) spectroscopy is a powerful technique for probing the chemical environment and electronic states of elements possessing NMR‐active nuclei in both liquid and solid phases. A recent study has demonstrated the robustness of the NMR technique in the characterization of Pt SACs. Monte Carlo simulations enable the transformation of NMR spectra into SAC‐specific features, allowing the coordination environment to be described with molecular‐level precision and thereby permitting quantitative evaluation of the distribution and uniformity of Pt sites [131]. Density function theory (DFT) simulation is an effective tool for simulating atomic and electronic structures of Sus‐SACs. It can be used to elucidate the interaction between SA sites and the support, as illustrated in Figure 8g.

## 5 Machine Learning‐Assisted Design, Rapid Precursor Screening, and Characterization of Sus‐SACs

Machine learning (ML) is increasingly reshaping materials research by enabling data‐driven discovery, predictive design, and intelligent characterization of catalysts. These capabilities are particularly relevant for Sus‐SACs, where the intrinsic heterogeneity of precursor materials presents a major challenge to controllable synthesis and scalable production. Recent advances in machine learning‐assisted spectroscopic analysis, have opened up new avenues for the rapid characterization and screening of diverse biomass and waste precursors. Such data‐driven techniques enable more efficient identification of promising raw materials for Sus‐SACs synthesis. Beyond precursor screening, ML‐assisted first‐principles calculations have created new opportunities for the rational design of SACs by enabling high‐throughput exploration of metal‐support interactions, prediction of stable coordination configurations, and identification of structure‐property relationships at the atomic scale. Meanwhile, ML‐driven analysis of spectroscopic and microscopic data is transforming catalyst characterization through automated feature extraction.

Despite these promising advances, the application of ML‐assisted strategies in the development of Sus‐SACs remains largely in its infancy. Accordingly, this section highlights emerging ML tools in the rapid Sus‐SACs precursor screening, SACs design, and structural characterization, with the aim of outlining prospective research directions that may accelerate the scalable and generalizable synthesis of Sus‐SACs.

### 5.1 Brief Introduction on Typical ML Models

Previous literature reviews have provided comprehensive introductions to various ML models and their features [135, 136, 137, 138]. Accordingly, this review will focus on a concise overview of the most frequently used models for the rapid characterization of biomass and solid waste.

K‐nearest neighbors (KNN) is a simple and intuitive non‐parametric machine learning algorithm applicable to both classification and regression tasks [139]. It predicts outcomes by locating the nearest data points in the feature space and inferring the label or value through majority voting (for classification) or averaging (for regression). Because KNN requires no explicit training phase, it can be computationally efficient for small datasets. However, its performance is highly sensitive to data scaling, the choice of distance metric, and the selection of the hyperparameter k which represents the number of neighbors considered in prediction. Moreover, KNN tends to suffer in high‐dimensional or large datasets due to the well‐known ‘curse of dimensionality.

Random forest (RF) is an ensemble learning method that constructs multiple decision trees using random subsets of the data and features, and aggregates their results through majority voting or averaging [140]. This bagging‐based approach enhances predictive accuracy and reduces overfitting compared to a single decision tree. RF is robust to noise, can handle both categorical and numerical variables, and provides measures of feature importance, which adds interpretability. Nevertheless, it may require substantial computational resources for very large datasets, and the resulting “forest” sacrifices some interpretability compared to individual trees.

Support vector machine (SVM) is a powerful supervised learning algorithm that seeks an optimal hyperplane to separate data points in a high‐dimensional space. It maximizes the margin between support vectors from different classes, thereby achieving strong generalization performance. SVM is especially effective for small to medium‐sized datasets with complex, nonlinear boundaries, where kernel functions (e.g., polynomial, radial basis function) can be applied. However, SVM can be computationally expensive for large datasets and sensitive to hyperparameters and kernel selection, both of which should be specified before training and require careful tuning. Moreover, SVM can be used for both regression (Support Vector Regression, SVR) and classification (SVCs) tasks [141].

Artificial neural networks (ANNs) are computational models inspired by the structure of biological neural systems, which is composed of interconnected layers of neurons [142]. ANNs are capable of capturing complex, nonlinear relationships between inputs and outputs, making them widely applicable to diverse problems such as image recognition, signal processing, and predictive modeling in chemistry and materials science. Variants such as convolutional neural networks (CNNs) and recurrent neural networks (RNNs) expand their scope to structured data and temporal sequences. While ANNs achieve high predictive accuracy, they are often criticized for their “black box” nature, high computational demands, and susceptibility to overfitting when training data are limited.

**FIGURE 9:**
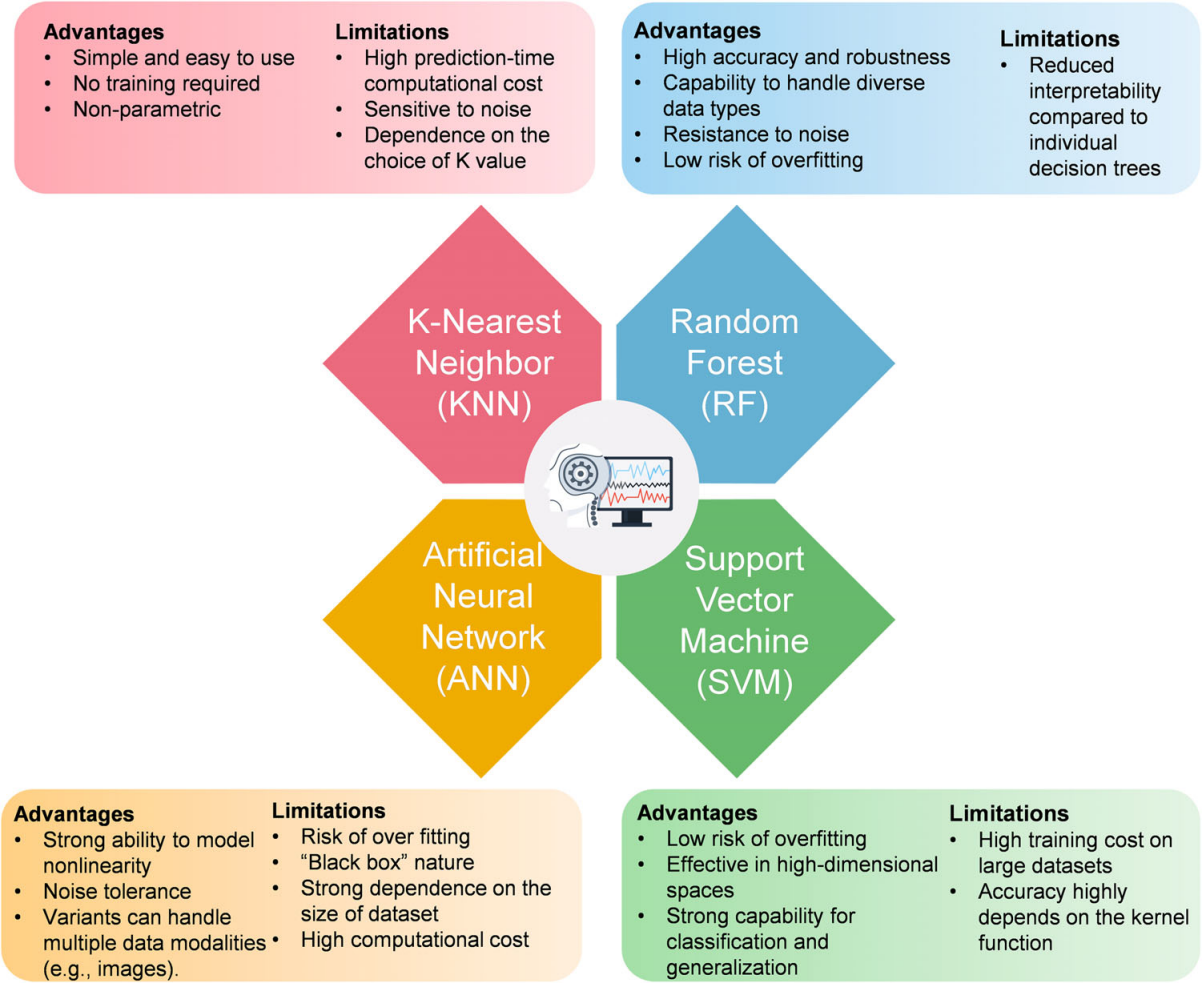
A summary of the characteristics of typical ML models.

The advantages and limitations of each ML model mentioned above are summarized in Figure 9. In practice, multiple models are often combined according to their respective strengths to ensure higher accuracy.

### 5.2 Applications of ML in Fast Characterizations of Biomass and Solid Waste Precursors

In the context of Sus‐SACs synthesis, the selection and characterization of precursors represent a critical factor that must be carefully considered, as the chemical composition and structural characteristics of the starting materials play a decisive role in determining the atomic dispersion, coordination environment, and electronic properties of the active sites in the resulting Sus‐SACs. As introduced above, precursors of Sus‐SACs are inherently diverse, containing different elemental compositions and mineral impurities in varying proportions. Moreover, the same biomass or solid waste precursors collected from different regions can vary significantly in composition, which hinders large‐scale production and broader application of Sus‐SACs. Therefore, systematic and rapid assessment of these multifactorial attributes is indispensable for guiding the rational design of Sus‐SACs. However, traditional approaches to evaluating biomass resources often suffer from inefficiency, inconsistent accuracy, and high experimental costs, largely due to the heterogeneous and complex nature of organic solid wastes. In recent years, ML techniques have proven highly effective in extracting valuable information from spectral data, offering unprecedented opportunities for the rapid characterization of biomass and solid waste [143].

**FIGURE 10:**
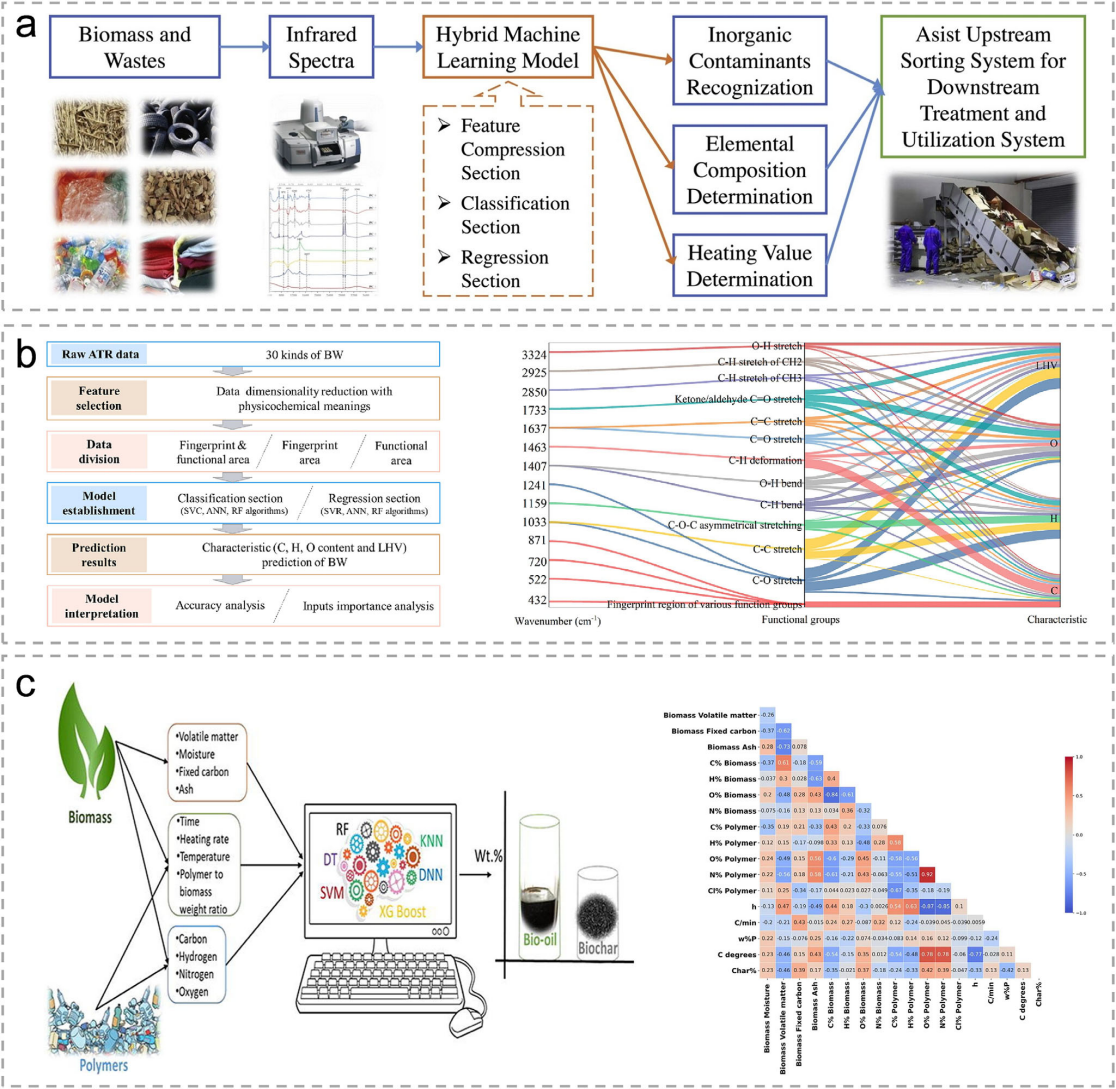
(a), Research framework of the fast characterization of biomass and waste by infrared spectra and machine learning models. Reproduced with permission [146]. Copyright 2020, Elsevier. (b), Research framework and the importance of features, functional groups on characterization of optimal C, H, O, and LHV prediction models. Reproduced with permission [147]. Copyright 2020, Elsevier. (c), Schematic diagram of a prediction model of the biochar and bio‐oil product via co‐pyrolysis of biomass waste and plastics. Reproduced with permission [152]. Copyright 2022, Elsevier.

Infrared (IR) spectroscopy is an analytical technique that characterizes materials by monitoring how they absorb or reflect infrared radiation [144]. It has become a mature non‐destructive approach for chemical compound identification and sorting. Additionally, the combination of IR spectroscopy with ML was widely studied for the fast classification and characterization of various biomass and solid waste [145]. Tao et al. developed a characterization and sorting system for biomass and solid waste by IR spectra integrated with multiple ML models, as shown in Figure 10a [146]. The raw IR spectra data were first processed by a feature compression section via primary component analysis (PCA) model to avoid overfitting. SVM models, including SVC and SVR, were employed to derive the final outcomes which can not only distinguish organic from inorganic fractions but also accurately predict elemental composition and heating value, achieving over 90% accuracy for C, O, and low heating value (LHV). In their recent work, they compressed the raw IR spectra data of 30 biomass or waste precursors such as bamboo leave, sewage sludge, and nylon cloth, etc. with physicochemical features that extracts high‐loading IR peaks tied to functional groups [147]. Meanwhile, their model quantitatively links bands/functional groups to the characterization of optimal C, H, O, and LHV prediction models. Figure 10b shows the research framework and the importance of functional groups on the characterization of optimal C, H, O, and LHV prediction models.

Raman spectroscopy is a widely used analytical technique in materials science, enabling both compositional analysis and rapid sample classification [148]. Recent studies have explored its integration with machine learning approaches to facilitate the preliminary characterization and categorization of raw materials, including carbon‐based substances and plastic wastes [138, 149]. Musu et al. combined PCA and SVM models for the accurate and robust classification of the plastics of polypropylene, polystyrene, and acrylonitrile‐butadiene‐styrene copolymer via Raman spectroscopy [150]. During the evaluation phase, the PCA‐SVM model exhibited outstanding accuracy and robustness, maintaining recognition rates above 95% even when the noise level was tripled.

Most precursors used in the synthesis of Sus‐SACs undergo high‐temperature carbonization. The resulting carbon materials critically influence the overall performance of Sus‐SACs, as they serve as the primary scaffold for anchoring metal atoms. Recent studies have employed ML approaches to predict biochar and bio‐oil products generated under different pyrolysis conditions, demonstrating the potential to rapidly estimate yield, structural characteristics, and elemental composition [151]. These advances open a promising pathway toward fast and reliable prediction of carbonized materials, thereby accelerating the rational design of Sus‐SACs. Alabdrabalnabi et al. integrated machine learning with experimental pyrolysis data to model the transformation of diverse biomass feedstocks [152]. By training predictive algorithms on key variables such as temperature, heating rate, and feedstock composition, their study achieved reliable forecasts of biochar and bio‐oil yield as well as carbon, nitrogen and hydrogen contents. The correlation analysis highlights the complex correlation among biomass composition, polymer characteristics, and process parameters, which jointly shape the yield and properties of pyrolysis‐derived carbon materials (Figure 10c).

### 5.3 Applications of ML in the Design, Synthesis, and Characterizations of SACs

**FIGURE 11:**
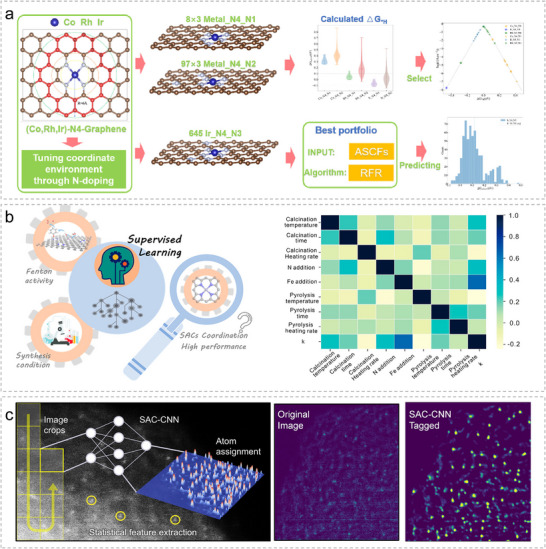
(a), Schematic illustration of the workflow for the ML‐assisted screening of (Co, Rh, Ir)‐N_4_ graphene SACs for HER. Reproduced with permission [157]. Copyright 2025, Elsevier. (b), ML‐assisted optimization of SAC synthesis parameters. Right: Heat map of the Pearson correlation coefficient matrix showing the correlations between key descriptors and the degradation rate (k) of a Fenton‐like catalyst. Reproduced with permission [159]. Copyright 2023, American Chemical Society. (c), ML‐assisted automated TEM image analysis for SAs detection. Reproduced with permission [161]. Copyright 2023, American Chemical Society.

A central goal in Sus‐SAC research is the rational design of atomically dispersed metal sites that combine structural stability with clearly defined active configurations and high catalytic efficiency. This objective is critically determined by three interrelated factors: the selection of support, the choice of metal sites, and the precise coordination environment engineering [153]. These factors collectively determine the electronic properties of the active sites, which govern their activity, selectivity, and stability across different reaction conditions. Density functional theory (DFT) calculations provide a fundamental platform for probing the intrinsic properties of SACs, such as electronic structure and adsorption energetics [154]. This capability creates opportunities for integration with ML tools, enabling high‐throughput screening, rational design, and the prediction of high‐performance Sus‐SACs [155]. Zhong et al. integrated ML with DFT calculations to perform high‐throughput screening of g‐C_3_N_4_‐supported single‐atom catalysts for the carbon dioxide reduction reaction (CO_2_RR) and hydrogen evolution reaction (HER) [156]. A systematic screening of 140 SACs@g‐C_3_N_4_ catalyst configurations was completed by the DFT‐ML approach, and 11 promising SACs with potential for efficient CO_2_RR and syngas production were identified. In Figure 11a, Wang et al. determined the HER activity of nitrogen‐doped (Co, Rh, Ir)‐N_4_‐graphene catalysts by DFT and ML methods [157]. DFT calculations were initially performed to evaluate the hydrogen adsorption free energy (ΔG_H*_) of 315 (Co, Rh, and Ir)‐N_4_ graphene‐based SACs. Among the evaluated configurations, Ir‐N_4_‐centered SACs were found to constitute a significant proportion of the most active structures. The combined ML‐DFT approach enables an approximately 200 000‐fold reduction in computational time compared to DFT‐only calculations.

For the synthesis of Sus‐SACs, the multitude of synthesis parameters, including support composition, pyrolysis temperature, and heating rate, etc., adds significant complexity to the formation of M‐N/C architectures. This hinders the establishment of definitive structure‐performance relationships as well as the controllable synthesis of Sus‐SACs. Yet, conventional catalyst development has largely relied on empirical trial‐and‐error approaches, resulting in slow experimental iteration and limiting the efficiency of research advancement [158]. Fu et al. established a machine‐learning‐based strategy to optimize synthesis conditions for Fenton‐like catalytic systems (Figure 11b) [159]. By screening a vast combinatorial space of synthesis conditions (10 sets of 10 synthesis parameters), the ML model predicted optimal parameters for maximizing Fenton activity in SACs. Experimental validation confirmed that 4 wt.% Fe loading, calcination at 640°C–680°C, and pyrolysis at 200°C–240°C produced highly active catalysts with minimal prediction error (±0.018 min^−1^). Under optimized conditions, the catalyst achieved a phenol degradation rate constant of 0.158 min^−1^, exceeding the performance of the initial dataset and outperforming most reported Fe‐based Fenton‐like catalysts‐thereby demonstrating the reliability of ML‐guided synthesis optimization. Meanwhile, machine learning offers a powerful approach to mitigating the environmental footprint of Sus‐SAC synthesis by enabling the identification of an optimal trade‐off among catalytic performance, structural stability, and sustainability. Through the systematic optimization of key synthesis parameters, such as the lowest effective pyrolysis temperature and the minimum required precursor concentration, ML can significantly reduce energy demand and material usage while maintaining the catalytic activity of Sus‐SACs.

Recent advances in machine learning have enabled data‐driven approaches for the characterization of Sus‐SACs, with TEM image analysis emerging as a critical route for extracting quantitative structural information. Although AC‐TEM is among the most powerful techniques for directly confirming atomic dispersion, its limited statistical representativeness, reproducibility challenges, and poor interoperability restrict its capacity to deliver comprehensive and robust characterization of these emerging catalytic materials [160]. To address this challenge, Mitchell et al. developed customized deep‐learning method (SAC‐CNN model) for automated atom detection in AC‐TEM image analysis as shown in Figure 11c [161]. Atomically dispersed Pt stabilized on functionalized carbon with complex 3D morphology represents a practically relevant test system with strong potential for thermo‐ and electrochemical applications. By detecting more than 20 000 atomic sites, the model facilitates comprehensive statistical analysis of structural parameters such as surface density, spatial proximity, clustering degree, and dispersion homogeneity. The customized neural network outperformed traditional image analysis approaches by reducing human bias via standardized atom‐labeling protocols while achieving roughly a 2000‐fold increase in processing speed per image.

**FIGURE 12:**
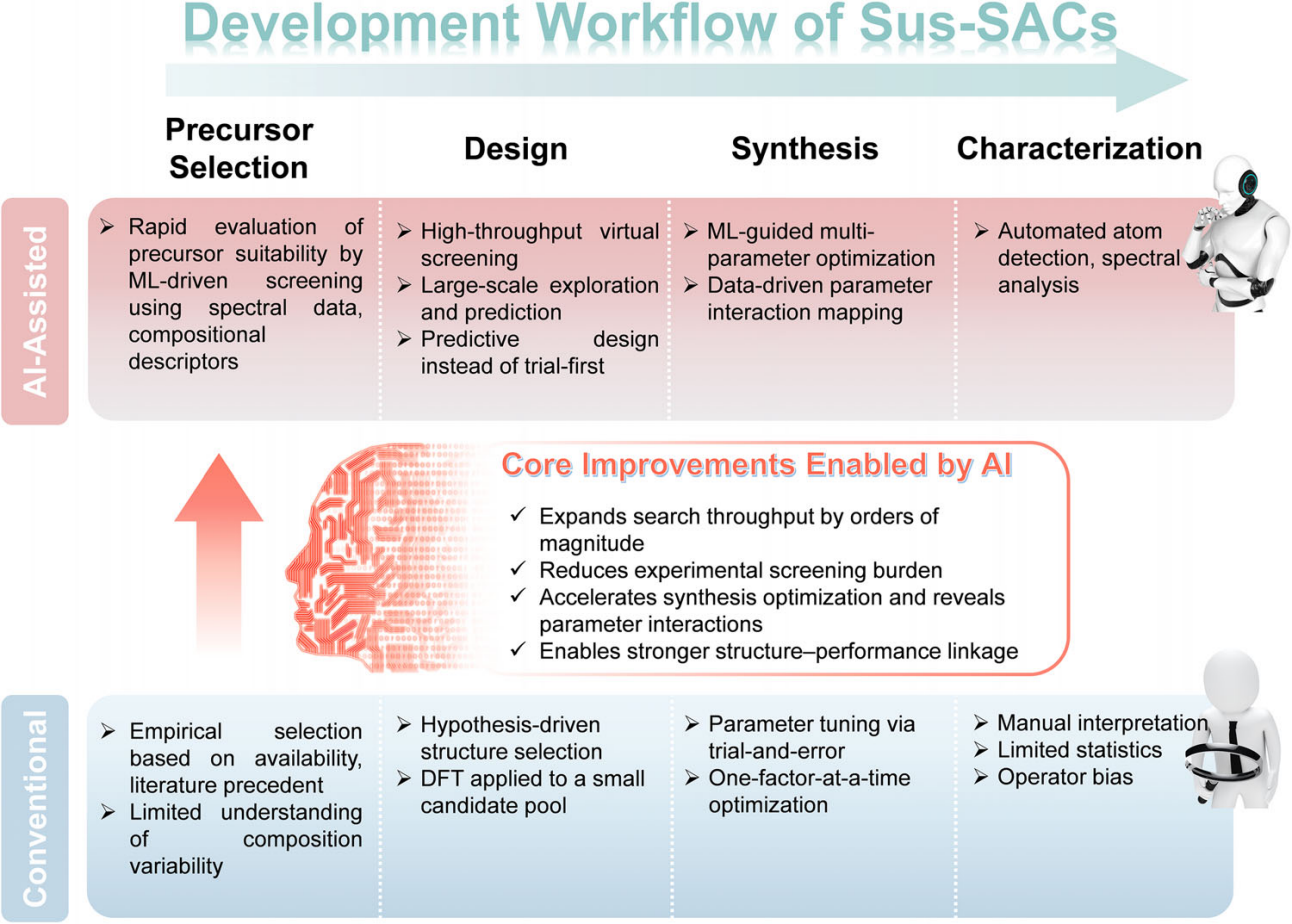
Comparison of conventional and AI‐assisted workflows for the development of Sus‐SACs.

As summarized in Figure 12, conventional Sus‐SAC development typically follows an empirical workflow through incremental trial‐and‐error. The characterization relies heavily on manual interpretation of microscopy and spectroscopy data. In contrast, AI‐assisted approach introduces a data‐driven loop across design, synthesis, and characterization, which enables high throughput screening, multi‐parameter synthesis optimization, and automated analysis of microscopy with reduced operator bias. Despite its significant promise, the effectiveness of AI‐assisted development of Sus‐SACs remains challenged by the need for high‐quality, standardized datasets and by uncertainties in model transferability across complex catalytic systems.

## 6 Applications of Sus‐SACs

The unique combination of atomically dispersed metal centers, abundant heteroatom functionalities, and diverse carbon architectures derived from biomass or solid waste precursors endows Sus‐SACs with exceptional activity, selectivity, and stability. As a result, Sus‐SACs have been increasingly explored in areas such as energy conversion, chemical synthesis and upgrading, as well as environmental remediation involving advanced oxidation processes and pollutant degradation. In this section, we will introduce the recent advances in the application of Sus‐SACs across these domains.

### 6.1 Sus‐SACs for Electrochemical Energy Conversion

#### 6.1.1 Applications of Sus‐SACs in HER

**FIGURE 13:**
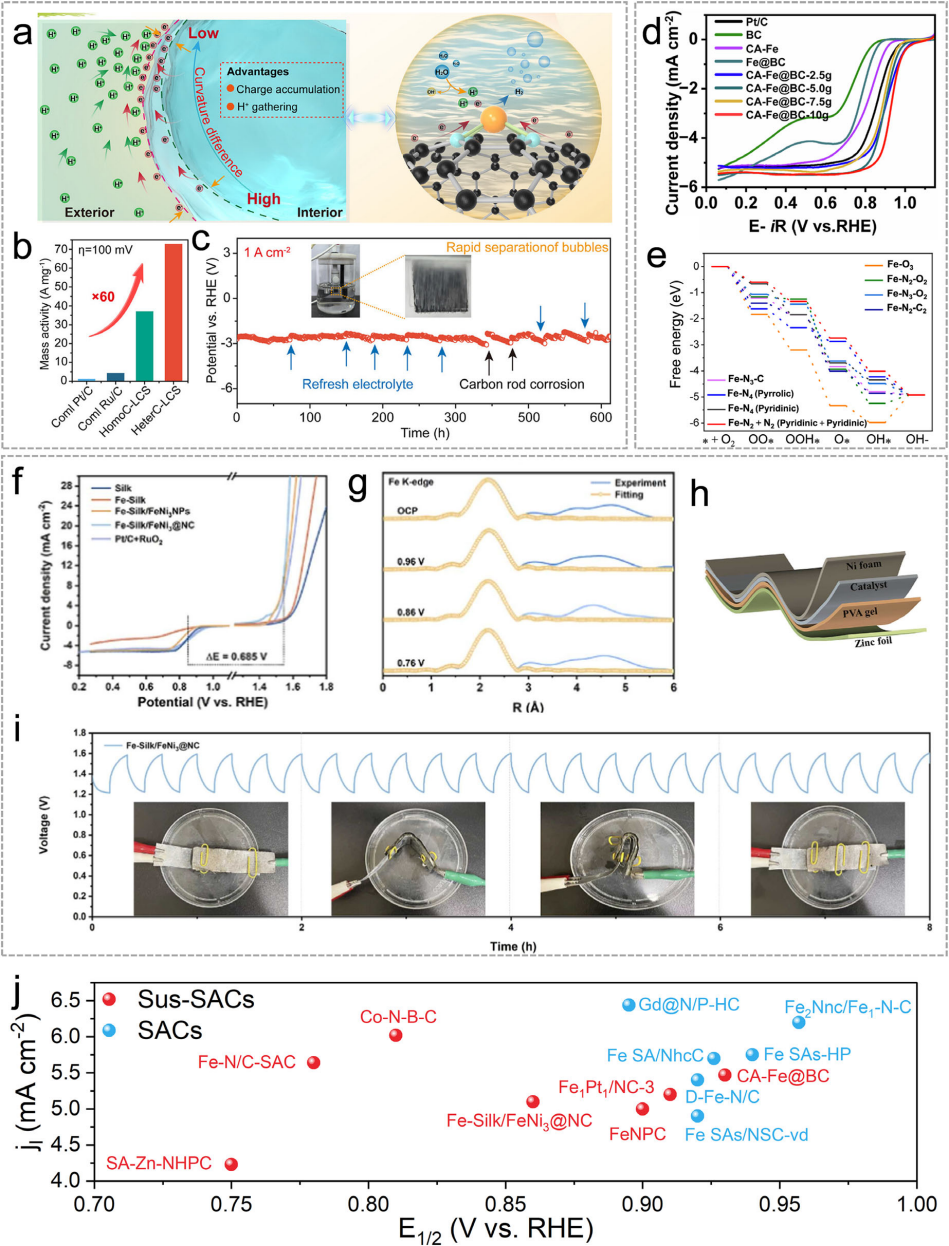
(a), Schematic diagram of the enhanced charge accumulation and H^+^ gathering on the heterogenous curvature interface. (b), mass activity of the Ru Sus‐SACs. (c), Stability test of the Ru Sus‐SACs. (a–c), Reproduced with permission [36]. Copyright 2025, Wiley‐VCH. (d), Linear sweep voltammetry (LSV) curves of the cellulose‐based Fe Sus‐SACs. (e), The free‐energy diagram of the reaction intermediates of ORR for the cellulose‐based Fe Sus‐SACs. (d‐e), Reproduced with permission [27]. Copyright 2025, the authors, Published by Springer Nature. (f), Overall polarization curves of the silk cocoon‐based Fe Sus‐SACs. (g), In situ EXAFS spectra of the silk cocoon‐based Fe Sus‐SACs during ORR. (h), Schematic diagram of the flexible ZAB assembled by the silk cocoon‐based Fe Sus‐SACs. (i), Stability test of the flexible ZAB assembled by the silk cocoon‐based Fe Sus‐SACs. (f–i), Reproduced with permission [115]. Copyright 2025, Elsevier. (j), A comparison of ORR performance between Sus‐SACs and other SACs. Sus‐SACs catalysts: Fe_1_Pt_1_/NC‐3 [165], CA‐Fe@BC [27], FeNPC [28], Fe‐N/C‐SAC [116], Co‐N‐B‐C [113], Fe‐Silk/FeNi_3_@NC [53], SA‐Zn‐NHPC [172]. Other SACs: D‐Fe‐N/C [173], Fe SA/NhcC [174], Fe2Nnc/Fe1‐N‐C [175], Fe SAs‐HP [176], Gd@N/P‐HC [177], Fe SAs/NSC‐vd [178].

Due to its high energy conversion efficiency, net‐zero footprint, and reliance on abundant water resources, HER is considered a clean, economical, and efficient pathway for future hydrogen production [162]. SACs, particularly those based on Pt‐group metals, have emerged as some of the most efficient electrocatalysts for the HER. In addition, carbon materials derived from biomass or waste provide an excellent platform for developing electrocatalysts and catalyst supports, owing to their favorable electrical conductivity and their adaptability for assembly into complex morphologies and microstructures for different reactions [163]. For HER, a catalysts with optimized curvature could induce enhanced local electric field effects around the active center which facilitates the rapid transfer of charges and reaction intermediates [164]. In recent work, Wang et al. proposed a strategy that integrates the advantages of SACs with the morphological tunability of biomass‐derived carbon [36]. Lignin, enriched with diverse functional groups, effectively anchors Ru SAs, and it is subsequently assembled into hollow spherical nanoreactors with various curvature differences. The heterogeneous curvature significantly enhances the accumulation of H^+^ at the catalytic interface while simultaneously accelerating the transport of H_2_O molecules to the active sites, as shown in Figure 13a. Moreover, DFT calculation demonstrates the optimized local electric field and the significantly reduced energy barrier of HER because of the nano‐curvature effect, leading to a low overpotential of 9 mV at a current density of 10 mA cm^−2^ (η_10_ = 9 mV), a high mass activity of 72.8 A mg^−1^ at 100 mV (Figure 13b) as well as long time stability over 600 h under 1 A cm^−2^ (Figure 13c). Another strategy to boost the catalytic performance of Sus‐SACs in HER is to introduce a second metal. Cao et al. synthesized a Fe/Pt dual metal Sus‐SACs from porphyra. The coordination environment for Pt atoms and Fe atoms within the catalyst are Pt‐N_4_ and Fe‐N_4_ respectively. The Pt/Fe dual metal Sus‐SACs exhibits high HER activity with an η_10_ of 27 mV [165].

#### 6.1.2 Applications of Sus‐SACs in Oxygen Electrocatalytic Reactions

Oxygen electrocatalytic reactions, including the oxygen reduction reaction (ORR) and oxygen evolution reaction (OER), are key processes in green energy technologies such as fuel cells, metal‐air batteries, and water electrolyzers [166, 167]. The ORR plays a central role in a variety of energy devices, including proton exchange membrane fuel cells (PEMFCs) and metal‐air batteries [168]. The ORR can proceed through two distinct pathways: a 2e^−^ route that yields H_2_O_2_ as the final product, and a 4e^−^ route that leads to the formation of water (H_2_O) [169]. In energy devices, the four‐electron transfer pathway is preferred over the two‐electron route, as it prevents the formation of corrosive peroxides and delivers higher energy efficiency [170]. Pt‐based SACs have been demonstrated efficient catalysts for ORR. For example, in Cao's work mentioned above, the Pt/Fe dual metal Sus‐SACs has a higher highest half‐wave potential (E_1/2_ = 0.91 V vs. RHE, reversible hydrogen electrode) than commercial Pt/C catalysts (E_1/2_ = 0.84 V vs. RHE) and the electron‐transfer number (*n*) remains in the range of 3.85–4 [165]. Recent studies have highlighted non‐precious metal SACs with M‐N‐C architectures (M = Fe, Co, Ni, etc.) as promising alternatives to costly Pt‐based catalysts [171]. Guo et al. synthesized an Fe Sus‐SACs with abundant Fe‐N_4_ coordination, which achieves high activity for pH‐universal ORR [27]. Their continuous activation strategy realizes a high Fe areal density of 4.7 atom nm^−2^ and a high specific surface area of 601.6 m^2^ g^−1^. Figure 13d shows the as‐obtained N‐rich Fe Sus‐SACs has a high pH‐universal ORR activity with a half‐wave potential of 0.93 V vs. RHE, 0.78 V vs. RHE, and 0.62 V vs. RHE in alkaline, acidic, and neutral electrolyte, respectively. DFT calculation results in Figure 13e further demonstrates the Fe‐N coordination effectively alters the potential‐determining step and lowers the energy barriers of ORR, thereby boosting the catalytic activity. A zinc‐air battery (ZAB) was assembled using the Fe Sus‐SACs as the cathode, and the assembly exhibited a high specific capacity of 792 mA·h·g_Zn_^−1^ and ultra‐long life span of over 650 h at 5 mA cm^−2^.

In rechargeable metal‐air batteries, the ORR during discharge and the OER during charging serve as the pivotal electrochemical steps governing device performance. Thus, bifunctional catalysts for both ORR and OER are preferable in metal‐air battery studies. Wang et al. developed a bifunctional Fe‐based Sus‐SACs using N‐doped carbon from silk cocoon as substrate [53]. Their Fe Sus‐SACs possesses two active sites, Fe SAs with Fe‐N_4_ coordination for ORR and FeNi_3_ nanoparticles for OER. It has a E_1/2_ of 0.86 V vs. RHE for ORR and an η_10_ of 315 mV for OER. Thus, the potential gap (ΔE) between E_1/2_ and E at η_10_ is only 0.65 V, as shown in Figure 13f. In situ EXAFS spectra in Figure 13g demonstrated the decrease of Fe‐Ni bond due to the adsorption of oxygen‐containing intermediates on the Fe active sites. Additionally, the Fe Sus‐SACs was assembled into a flexible all‐solid‐state ZAB as shown in Figure 13h, and the as‐obtained ZAB has a specific capacity of 705 mA h g^−1^. The ZAB assembly can stably operate for 30 h (Figure 13i). Figure 13j compares the ORR performance of Sus‐SACs and state‐of‐the‐art SACs using E_1/2_ and limiting current density (j_l_) as the primary performance descriptor. Most catalysts display similar diffusion‐limited current densities. In contrast, variations in E_1/2_ more clearly differentiate catalytic performance. The E_1/2_ values of advanced SACs are predominantly distributed within 0.90–0.98 V vs. RHE. Encouragingly, several Sus‐SACs exhibit E_1/2_ values approaching those of leading SAC systems. This comparison suggests a transition of Sus‐SACs from alternative materials toward fully competitive ORR catalysts.

#### 6.1.3 Applications of Sus‐SACs in Electrochemical CO_2_RR

The extensive use of fossil fuels has led to a rapid increase in atmospheric carbon dioxide (CO_2_) levels, contributing to climate change and global energy challenges [179]. The electrochemical CO_2_RR conducted under ambient conditions and powered by renewable energy resources not only mitigates the adverse effects of excessive CO_2_ emissions but also enables the production of value‐added carbon‐based fuels, making it one of the most promising strategies for carbon utilization [180, 181]. Carbon monoxide (CO) is often targeted as the primary product of CO_2_ electroreduction because it can be produced efficiently via a low‐energy, two‐electron pathway and serves as a versatile platform intermediate for scalable carbon‐neutral fuel and chemical synthesis [182].

**FIGURE 14:**
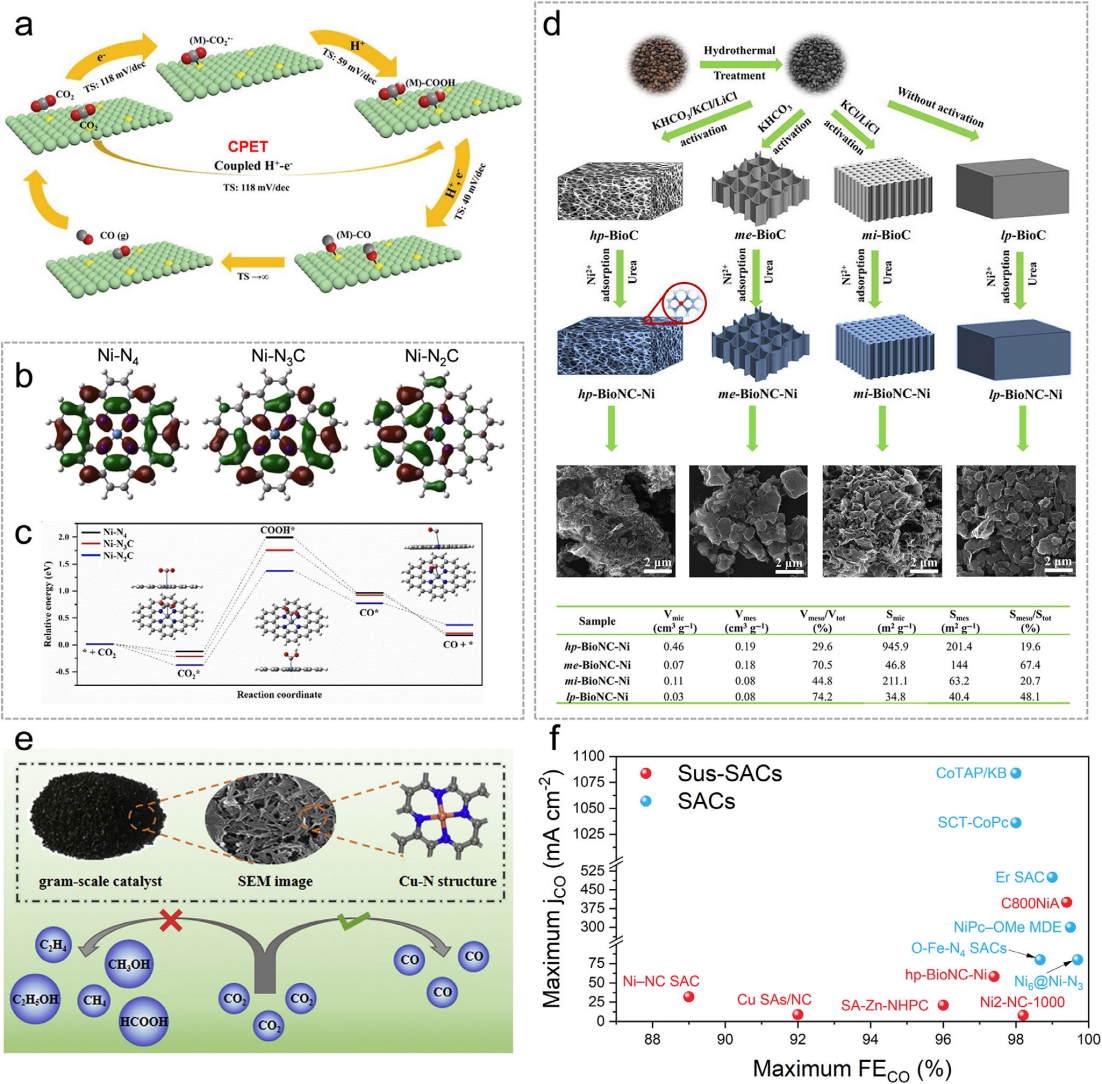
(a) Reaction pathway of the electroreduction of CO_2_ to CO and the estimated Tafel slope (TS) values for each possible rate‐determining step. Reproduced with permission [181]. Copyright 2020, Wiley‐VCH. (b), Calculated electron distribution of HUMO. (c), Calculated free energy diagrams for CO_2_RR with different Ni‐N coordination environment. (b‐c), Reproduced with permission [187]. Copyright 2023, Wiley‐VCH. (d), Synthetic scheme, morphology, and the porosity of p‐BioNC‐Ni. Reproduced with permission [188]. Copyright 2023, American Institute of Chemical Engineers. (e), Schematic diagram of the synthesis of Cu‐based Sus‐SACs for CO_2_RR. Reproduced with permission [189]. Copyright 2020, Elsevier. (f), A comparison of CO_2_RR performance between Sus‐SACs and other SACs. Sus‐SACs catalysts: Ni‐NCSAC [190], Cu SAs/NC [189], SA‐Zn‐NHPC [172], hp‐BioNC‐Ni [188], Ni_2_‐NC‐1000 [187], C800NiA [186]. SACs catalysts: CoTAP/KB [191], SCT‐CoPc [192], Er SACs [193], NiPc‐Ome MDE [194], O‐Fe‐N4 SACs [195], Ni_6_@Ni‐N_3_ [196].

The electrochemical CO_2_RR generally involves three key stages. The first stage is the adsorption of CO_2_ molecules onto the catalyst surface. Subsequently, the adsorbed CO_2_ species undergo proton‐electron transfer to form reaction intermediates. In the final stage, the reduced products desorb from the catalyst surface [183]. The reaction mechanism of CO_2_‐CO is depicted in Figure 14a. The ^*^COOH intermediate can be formed either through one‐step via a concerted proton–electron transfer (CPET) mechanism or through a two‐step proton‐decoupled electron transfer process where ^*^COOH is formed through the ^*^CO_2_^−^ intermediate [184]. The ^*^COOH intermediate is subsequently reduced to ^*^CO through an additional proton–electron transfer, followed by the desorption of weakly bound ^*^CO to form CO. SACs featuring porphyrin‐like or phthalocyanine‐inspired M‐N‐C coordination structures have demonstrated high activity and excellent CO selectivity for the electrochemical CO_2_‐CO conversion. Zhang et al. dispersed nickel phthalocyanines on carbon nanotubes and achieved an outstanding selectivity (>99.5%) at high current densities of up to ‐300 mA cm^−2^ [185]. In recent work, Liu et al. integrated AI‐powered large‐scale data mining and identified cobalt‐phthalocyanine (CoPc) as a promising candidate for CO_2_‐CO electroreduction. Meanwhile, they broke the conventional chemical intuition which attributed the catalytic performance to single‐layer Pc/carbon structure. They uncovered a multilayer CoPc core–shell architecture supported on Ketjen Black, where surface charge transfer plays a key role in dramatically boosting electrocatalytic CO_2_‐CO performance.

Owing to the sustainability and low‐cost features of biomass‐ and solid‐waste‐derived precursors, the application of Sus‐SACs in in electrochemical CO_2_RR represents a promising and environmentally responsible strategy for CO_2_ utilization. Ni‐based Sus‐SACs have been widely reported for CO_2_‐CO. The Ni‐based Sus‐SACs synthesized by Abbas et al. delivered a current density of ‐300 mA cm^−2^ with an outstanding CO Faradaic efficiency (FE_CO_) of 99.4% at an overpotential of 235 mV [186]. The study from Norinaga's group revealed that the CO_2_RR performance of Ni‐based Sus‐SACs is strongly influenced by the coordination environment of the Ni single‐atom active sites [187]. Ni Sus‐SACs derived from lignin with different coordination structures (Ni‐N_4_, Ni‐N_3_C, and Ni‐N_2_C) were obtained by manipulating the thermal activation. Among three Ni‐SA configurations, Ni‐N_2_C achieved the highest FE_CO_ of 98.2 % at ‐0.9 V vs. RHE. DFT calculation in Figure 14b demonstrated that the highest occupied molecular orbital (HOMO) on the Ni atom was entirely different depending on the Ni‐N_x_ coordination environment. For low Ni‐N coordination numbers, the HOMO is predominantly localized on the central Ni atom. This localized electron density renders the Ni center more favorable for initial CO_2_ adsorption and facilitates electron transfer during the reaction process. Consequently, the Ni‐N_2_C structure exhibits the lowest Gibbs free energy for CO_2_ adsorption and the smallest free energy barrier for the rate‐determining step in the CO_2_‐to‐CO conversion pathway as shown in Figure 14c. Porosity engineering is another effective strategy for enhancing the CO_2_RR performance of Ni‐based Sus‐SACs. Zhang et al. synthesized biochar‐derived single‐atom Ni catalysts with tunable porosity (p‐BioNC‐Ni), where “p” denotes the pore type, including hierarchically porous (hp), mesoporous (me), microporous (mi), and less porous (lp) structures as depicted in Figure 14d [188]. This study demonstrated that hierarchical porosity can significantly enhance the CO_2_RR efficiency. The hierarchically porous catalyst (hp‐BioNC‐Ni) achieved a FE_CO_ of 97.4% at ‐1.2 V vs. RHE.

Beyond Ni‐based Sus‐SACs, other transition metals, such as Zn and Cu, have also been reported for CO_2_RR application. Yang et al. developed a scalable strategy for the gram‐scale synthesis of Cu‐based Sus‐SACs via a two‐step carbonization as shown in Figure 14e [189]. The Cu‐based Sus‐SACs with Cu‐N_4_ coordination exhibited high FE_CO_ of 92% at ‐0.7 Vs. RHE and stability over 30 h. DFT calculation revealed that the formation of ^∗^COH at Cu‐N_4_ site requires the highest free‐energy barrier (2.83 eV), followed by ^∗^COCO (1.04 eV) and ^∗^CHO (0.87 eV). In contrast, the release of ^∗^CO to generate CO is energetically favorable. These findings are consistent with experimental observations indicating that CO_2_ preferentially converts to CO with high selectivity. Wang et al. synthesized Zn‐based Sus‐SACs from apples and egg whites [172]. Experimental studies reveal that the Zn‐N_4_ sites in SA‐Zn‐NHPC govern its outstanding CO_2_RR performance, achieving a FE_CO_ of 96% at an overpotential as low as 0.33 V. Figure 14f shows the comparison of the CO_2_RR performance between Sus‐SACs and other state‐of‐art SACs in terms of FE_CO_ and j_CO_. Notably, Sus‐SACs deliver CO selectivity comparable to leading SAC systems, indicating that the use of sustainable precursors does not inherently compromise catalytic performance. However, their current densities generally remain below those of the best‐performing catalysts. This discrepancy may partly arise from mass‐transport limitations associated with the widespread use of H‐cell configurations rather than insufficient intrinsic activity. Consequently, future research on Sus‐SACs should move beyond activity‐oriented evaluations in H‐cells toward demonstrations in industrially relevant flow electrolyzers, thereby bridging the gap between sustainable catalyst design and practical CO_2_ electroreduction.

### 6.2 Sus‐SACs for Chemical Synthesis and Upgrading

**FIGURE 15:**
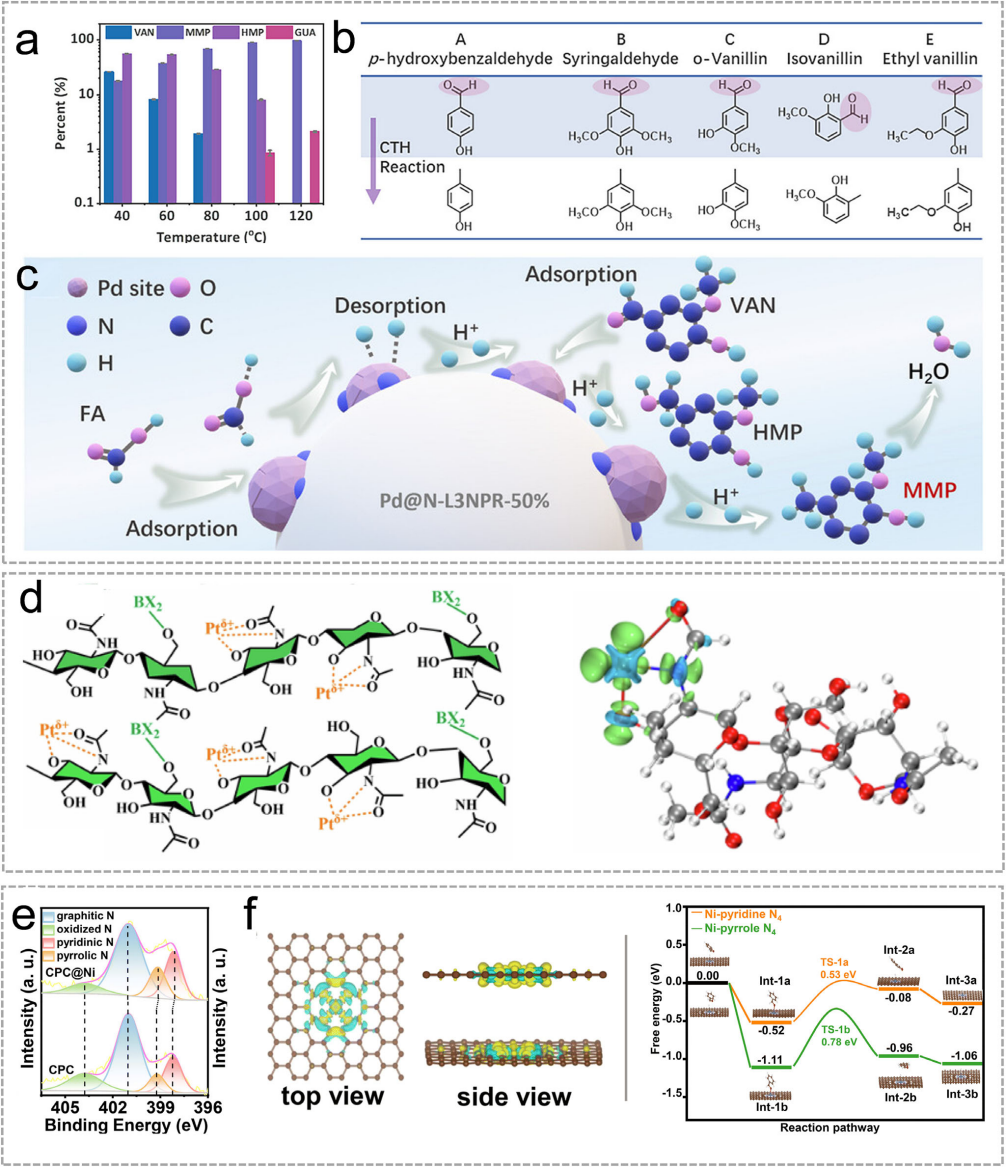
(a), Catalytic performance of Pd‐based Sus‐SACs for VAN CTH. (b), CTH reaction of lignin‐derived aldehydes by the Pd‐based Sus‐SACs. (c), The schematic diagram of the mechanism for the VAN CTH reaction over the Pd‐based Sus‐SACs. (a–c), Reproduced with permission [35]. Copyright 2025, Wiley‐VCH. (d), Structure diagram of the Pt‐based Sus‐SACS derived from chitin. Reproduced with permission [46]. Copyright 2025, the authors, Published by Springer Nature. (e), XPS spectra analysis for the N species within the Ni‐based Sus‐SACS. (f), Illustration for the Coordinative Structure of the Ni‐based Sus‐SACs (left) and the reaction pathway and corresponding Gibbs free energy profiles for benzyl alcohol oxidation by using the Ni‐pyridine‐N4 and Ni‐pyrrole‐N4 models (right). (e‐f), Reproduced with permission [47]. Copyright 2025, the Royal Chemical Society.

Catalytic transfer hydrogenation (CTH) of lignin‐derived aldehydes refers to the efficient catalytic reduction of aldehydes obtained from lignin depolymerization using a hydrogen donor instead of gaseous hydrogen, producing corresponding alcohols or upgraded bio‐based chemicals [197, 198]. Li et al. developed a Ni Sus‐SACs via the pyrolysis of lignin in the presence of melamine [34]. During synthesis, Ni atoms were initially anchored by negatively charged functional groups such as phenolic ‐OH and subsequently coordinated with nitrogen, forming Ni‐N_3_ structures as the active sites. The resulting Ni Sus‐SACs exhibited excellent performance in the catalytic transfer hydrogenation (CTH) of vanillin (VAN) using formic acid (FA) as the hydrogen donor, achieving 98.93% VAN conversion and 97.3% selectivity toward 2‐methoxy‐4‐methylphenol (MMP) at 180°C. It is noteworthy that the interaction between the SA sites and the support can synergistically enhance the catalytic performance. Pang et al. proposed a non‐carbonization strategy to synthesize Sus‐SACs with Pd atomically dispersed on lignin‐functionalized phenolic resin (LPR) nano‐chain‐like morphology [35]. The LPR support contains high ‐NH_2_ groups, which are essential for the immobilization of Pd atoms under room temperature. The unique support architecture combined with atomically dispersed Pd active sites enabled the Pd Sus‐SACs to selectively adsorb and reduce the –CHO groups in lignin‐derived aldehydes during CTH, using FA as the hydrogen donor under mild conditions (80°C) with approximately 100% VAN conversion and 97.91% MMP selectivity, as shown in Figure 15a. The catalyst also exhibited superior reactivity across a range of lignin derivatives, including o‐vanillin, syringaldehyde, isovanillin, ethyl vanillin, and p−hydroxybenzaldehyde (Figure 15b). DFT calculations further revealed that the abundant –NH_2_ groups on the catalyst surface enhance the adsorption of both FA and ‐CHO species. Moreover, the favorable Pd‐N coordination environment significantly reduces the energy barrier for the CTH of VAN as depicted in Figure 15c.

Chemo‐selective hydrogenation is widely used to produce value‐added chemicals, pharmaceutical intermediates, fragrances, and stabilized biomass‐derived products, making it a key transformation in both fine chemical synthesis and sustainable molecular upgrading [199]. Heterogeneous catalysts, while offering long operational lifetimes, often lack the ability to precisely control hydrogenation selectivity. The rational design of the catalytic environment of SACs is therefore crucial, requiring both flexibility and robustness within the local microenvironment surrounding the SA sites. Li et al. developed a simple and economical strategy to prepare chitin‐supramolecular–stabilized single‐atom Pt catalysts by exploiting strong coordinative interactions between the metal and the organic polymer network [46]. The Pt single atoms are coordinated with the organic supramolecular ligand‐like framework, which provides a flexible coordination environment reminiscent of homogeneous catalysts while maintaining the robustness characteristic of heterogeneous systems as shown in Figure 15d. Using the Pt Sus‐SACs, the study enables tunable hydrogenation across 31 substrates to afford 62 products in yields of up to 99%, with outstanding chemo‐selectivity (>90:1) and an impressive turnover number (TON) of 121350.

N‐Alkylation is a highly important transformation in organic synthesis, and the resulting N‐alkylamines are widely used in pharmaceuticals, health products, additives, and functional materials. Zhu et al. synthesized a Ni Sus‐SACs from wasted chitin, which offers both physical and chemical confinement to firmly anchor Ni atoms through its abundant micropores and intrinsic N dopants [47]. This Ni‐based Sus‐SACs possesses typical Ni‐N_4_ coordination, and it exhibited excellent catalytic performance in N‐alkylation of alcohols and amines with a 99% yield at 120°C and a high turnover frequency (TOF) of 11.79 h^−1^. The reactivity remains satisfactory after 8 cycles. Structural analysis revealed the N species are mainly pyridine‐N and pyrrole‐N as shown in Figure 15e. DFT calculation demonstrated that the Ni–pyridine–N_4_ configuration was the dominant active site, providing a more favorable environment for cleaving the C‐H bond in Ph‐CH_2_O, thereby promoting alcohol dehydrogenation and driving the overall reaction forward (Figure 15f).

### 6.3 Sus‐SACs for Water Pollution Treatment

Environmental degradation has become one of the defining challenges of the 21st century, posing serious threats to global ecological stability and socioeconomic development [200]. Among various forms of environmental degradation, water pollution has drawn the greatest concern. Rapid industrialization and unsustainable consumption patterns have further intensified the depletion and contamination of already limited freshwater resources [201, 202]. In modern society, o Water Soluble Organic Pollutants, oils, microplastics, and heavy metals constitute the major contaminants found in water systems [203, 204]. Among these, organic pollutants represent the largest category. Fenton and Fenton‐like reactions, which generate highly reactive oxidative species (e.g., peroxymonosulfate (PMS), persulfate (PDS), and H_2_O_2_), have been widely employed in environmental remediation [205, 206]. SACs overcome the size limitations of conventional metal particles and achieve nearly 100% metal atom utilization, thereby exhibiting exceptional Fenton‐like activity. Their low‐coordination environments endow the single atoms with high surface energies, which help lower the activation barriers for peroxide activation. Moreover, the strong interactions between the metal atoms and the support enhance electron transfer within the Fenton‐like system, further improving catalytic performance [207]. In this section, we highlight recent advances in the use of Sus‐SACs for Fenton‐like reactions aimed at pollutant removal.

**FIGURE 16:**
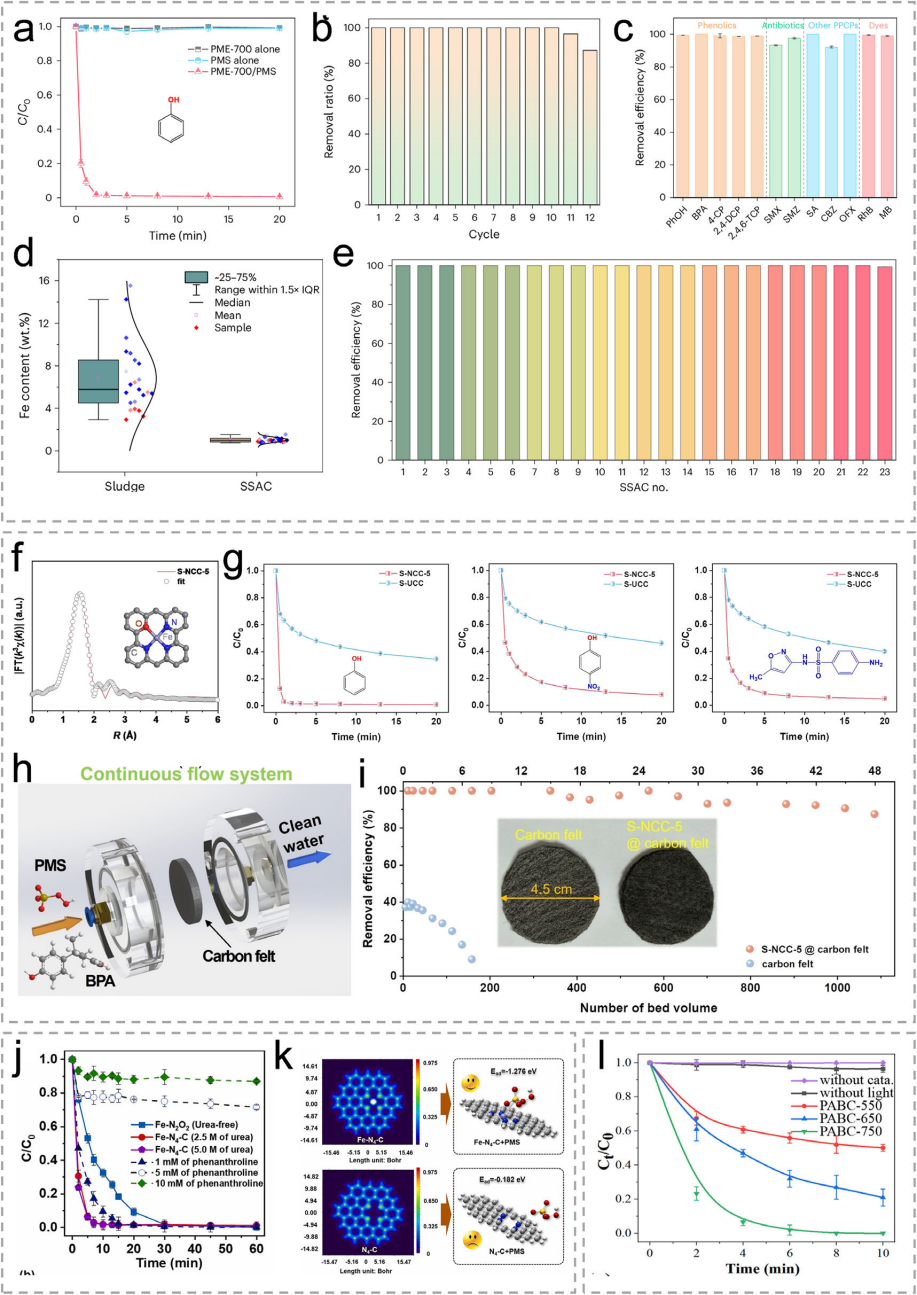
(a), Phenol removal on Fe‐based Sus‐SACs. (b), Reusability of Fe‐based Sus‐SACs. (c), Removal efficiency of various pollutants following 20 min reactions in a Fe‐based Sus‐SACs. (d), Fe content distribution in 23 Fe‐based Sus‐SACs and their sludge precursors. (e), Removal efficiency of phenol following 20 min reactions of 23  Fe‐based Sus‐SACs. (a–e), Reproduced with permission [65]. Copyright 2024, the authors, Published by Springer Nature. (f), EXAFS spectra and fitting curves in R space of nano‐confined Fe Sus‐SACs. (g), Comparison of activities of nano‐confined Fe Sus‐SACs and unconfined catalysts for degradation of different pollutants. (h), Schematic diagram of the assembled continuous flow system. (i), Long‐term test of BPA degradation in the continuous flow system. (f–i), Reproduced with permission [66]. Copyright 2025, Wiley‐VCH. (j), Catalytic performances of Fe‐based Sus‐SACs derived from Enteromorpha. (k), electron density distribution of Fe‐N_4_‐C and N_4_C, and the PMS adsorption ability on them. (j,k), Reproduced with permission [58]. Copyright 2023, Elsevier. (l), Catalytic performance of Mn‐based Sus‐SACs for RhB degradation. Reproduced with permission [63]. Copyright 2022, American Chemical Society.

Gu et al. upcycled municipal sewage into a Fe‐based Sus‐SACs as Fenton‐like catalysts for water purification [65]. EXAFS spectra revealed that the coordination of Fe within the catalyst is mainly Fe‐N_4_. The resulting Fe Sus‐SACs showed outstanding Fenton‐like catalytic activity toward phenolic pollutants, achieving ultrafast degradation kinetics even at a low oxidant‐to‐pollutant ratio of 3.0. As shown in Figure 16a, more than 99% phenol was removed within 2.0 min. The resulting catalyst also delivered a high TOF of 372.52 h^−1^ and maintained excellent activity over ten consecutive catalytic cycles (Figure 16b). Furthermore, the sewage‐derived Fe Sus‐SACs exhibited broad catalytic applicability, effectively degrading a range of water‐soluble pollutants from phenolics to dyes with high decontamination efficiency, as illustrated in Figure 16c. Impressively, the strategy proved to be highly universal. As shown in Figure 16d, Fe Sus‐SACs with comparable structural features were successfully synthesized from wastewater sewage collected across 23 regions in China, despite the variations in their chemical compositions. Figure 16e shows that these catalysts all exhibited superior catalytic activity with a removal efficiency over 99%. In their recent work, the silica and alumina components within the sewage were utilized to confine Fe SAs [66]. Confinement strategy effectively prevented the aggregation of Fe atoms. In addition, the coordination structure of the Fe centers was modulated to form an Fe‐N_3_O as shown in Figure 16f. Compared with unconfined catalysts, the confined Fe Sus‐SACs exhibited much higher reactivity for the removal of phenol, 4‐nitrophenol (4‐NP), and sulfamethoxazole (SMX) as shown in Figure 16g. In the nanoconfined Fe Sus‐SACs, the Fe‐N_3_O coordination environment plays a decisive role in modulating the interfacial energy and electronic interactions among the catalyst, the oxidant, and the pollutant. This tailored coordination structure optimizes chemical complexation, promotes structural activation of the reactants, and governs the formation of reactive intermediates, thereby shaping the overall reaction mechanism. Additionally, the catalyst was assembled into a commercial carbon felt filter for the demonstration of practical application (Figure 16h). Figure 16i shows that the filter sustained a high bisphenol A (BPA) removal efficiency of 87.5% during 48 h of continuous operation, treating a total of 1087 bed volumes.

Naproxen (NPX), a widely used non‐steroidal anti‐inflammatory drug, has become an emerging contaminant in water resources. Qi et al. developed a Co‐based Sus‐SACs from lignin as catalysts for the NPX degradation [208]. The catalyst with Co‐N_4_ sites delivered a high reaction activity to completely degrade NPX within 1 h. Additionally, the Co‐ based Sus‐SACs are efficient in degrade other contaminants like paracetamol (PCM), ciprofloxacin (CIP), bisphenol A (BPA). Recent studies showed that the coordination of M‐N‐C structure (M: Fe, Co, Mn, etc.) enhances charge transfer and stability and thus promotes the activity [16]. Yin et al. utilized the rich Fe content in Enteromorpha for the synthesis of Fe Sus‐SACs with Fe‐N_4_‐C configuration. In addition, the introduction of urea promoted the formation of Fe‐N_4_‐C sites, whereas the absence of urea led predominantly to the generation of Fe‐N_2_O_2_ coordination structures. Compared with the catalyst of Fe‐N_2_O_2_ configuration, Fe Sus‐SACs with Fe‐N_4_‐C exhibited faster and higher naproxen (NPX) removal efficiency as shown in Figure 16j. Figure 16k for the DFT results shows that the Fe center in Fe‐N_4_‐C configuration exhibited higher adsorption ability toward PMS. Strong PMS adsorption enhances electron transfer, lowers activation barriers, and thereby significantly improving catalytic performance.

The incorporation of light irradiation into conventional Fenton chemistry significantly promotes the formation of reactive oxygen species, thereby establishing a photo‐Fenton process with substantially enhanced catalytic efficiency. Cui et al. reported a Mn‐based Sus‐SACs from a Mn hyperaccumulator, P. americana as photocatalyst for the degradation of RhB [63]. As shown in Figure 16l, the Mb‐based Sus‐SACs delivered a degradation rate reached ∼100% within 10 min. Moreover, the degradation rate remains above 90% after 6 cycles of experiment, demonstrating its good stability. Combine with in situ XANES and DFT calculation, Mn‐N_4_ sites are the dominant active center for photo‐Fenton reaction.

Table 2 summarizes the applications of Sus‐SACs along with their coordination configurations and key performance parameters.

**TABLE 2:** A summary of the main applications of Sus‐SACs and their key performance parameters.

Application	Sus‐SACs	Raw Sources	Single Atom	Coordination	Reaction	Peformance	Reference
Electrocatalysis	HeterC‐LCS	Lignin	Ru	Ru‐N_4_	HER	Apparent Activity: η_10_ = 9 mV Mass Activity: 72.8 A mg^−1^ at 100 mV Tafel Slope: 8.7 mV dec^−1^ Over 600 h Stability at 1A cm^−2^	[36]
	Fe_1_Pt_1_/NC‐3	Porphyra	Pt & Fe	Pt‐N_4_ Fe‐N_4_	HER	Apparent Activity: η_10_ = 27 mV Tafel Slope: 28 mV dec^−1^	[165]
					ORR	E_1/2_ = 0.91 V vs. RHE n = 3.85‐4
	CA‐Fe@BC	Bacteria Cellulose	Fe	Fe‐N_4_	ORR Zn‐Air Batteries	E_1/2_ = 0.93 V vs. RHE (Alkaline) E_1/2_ = 0.78 V vs. RHE (Acidic) Specific Capacity of 792 mA·h·g_Zn_^−1^ Life span over 650 h at 5 mA cm^−2^	[27]
	FeNPC	Cellulose	Fe	Fe‐N_3_P	ORR ZAB	E_1/2_ = 0.90 V vs. RHE Maximum power density (P_max_) = 149.4 mW cm^−2^ Specific Capacity of 761.8 mA·h·g_Zn_^−1^	[28]
	Fe‐N/C‐SAC	Pork Liver Powder	Fe	Fe‐N_4_	ORR	E_onset_ = 0.89 V vs. RHE n = 3.9‐4	[116]
	Co‐N‐B‐C	Chitosan	Co	Co–N	ORR	E_1/2_ = 0.81 V vs. RHE Limiting current density (J_L_) = 6.02 mA cm^−2^ P_max_ = 137.4 mW cm^−2^	[113]
	FeSAs‐NiCo alloy@N‐C sphere	Chitosan	Fe	Fe‐N_4_	ORR	E_onset_ = 1.08 V vs. RHE n = 3.42	[112]
	Fe‐Silk/FeNi_3_@NC	Silk Cocoon	Fe	Fe‐N_4_	ORR ZAB	E_1/2_ = 0.86 V vs. RHE ΔE = 0.65 V Specific Capacity of 705 mA·h·g^−1^ (all‐solid ZAB)	[53]
					OER	η_10_ = 315 mV
	C800NiA	Adenine	Ni	Ni‐N_4‐x_C_x_	CO_2_RR	Maximum Faradaic efficiency (Max FE_CO_) = 99.4% at −0.235 V vs. RHE Maximum Partial current density for CO (Max j_CO_) = −400 mA cm^−2^	[186]
	Ni_2_‐NC‐1000	Lignin	Ni	Ni‐N_2_C	CO_2_RR	Max FE_CO_ = 98.2% at −0.9 V vs. RHE Max j_CO_ = −8 mA cm^−2^	[187]
	hp‐BioNC‐Ni	Biochar	Ni	Ni‐N_4_	CO_2_RR	Max FE_CO_ = 97.4% at −1.2 V vs. RHE Max j_CO_ = −58.2 mA cm^−2^	[188]
	Ni–NC SAC	Glucose	Ni	Ni‐N_x_	CO_2_RR	Max FE_CO_ = 89% at −0.85 V vs. RHE Max j_CO_ = −35 mA cm^−2^	[190]
	Cu SAs/NC	Chitosan	Cu	Cu‐N_4_	CO_2_RR	Max FE_CO_ = 92% at −0.7 V vs. RHE Max j_CO_ = −8.9 mA cm^−2^ Stability over 30 h	[189]
	SA‐Zn‐NHPC	Apples and egg white	Zn	ZnN_4_	CO_2_RR	Max FE_CO_ = 96% at −0.33 V vs. RHE Max j_CO_ = −21 mA cm^−2^	[172]
Chemical Synthesis and Upgrading	Ni SAs‐N@LC	Lignin	Ni	Fe‐N_3_	VAN CTH	98.93% VAN conversion (180°C) 97.3% selectivity toward MMP (180°C) Conversion and selectivity remained above 95% after 4 cycles	[34]
	Pd@N‐L3PR‐50%	Lignin	Pd	Pd‐N_3_	VAN CTH	∼100% VAN conversion (80°C) 97.91% selectivity toward MMP (80°C) High reactivity for a range of lignin derivatives Satisfactory recycling stability over 5 cycles	[35]
	SS‐Pt‐CSNs	Chitin	Pt	Pt‐N/O_3_	Chemo‐selective hydrogenation	Enables tunable hydrogenation across 31 substrates to afford 62 products Yield up to 99% Chemo‐selectivity >90:1 TON = 121,350	[46]
	CPC@Ni	Chitin	Ni	Ni‐N_4_	N‐alkylation of alcohols and amines	Yield up to 99% at 120°C Satisfactory recycling stability over 8 cycles TOF = 11.79 h^−1^	[47]
Environmental Remediation	PME‐700	Waste Water Sewage	Fe	Fe‐N_4_	Fenton‐like Reaction	Above 99% phenol removal efficiency in 2 min Applicable to a variety of pollutants Stable for more than 10 cycles	[65]
	S‐NCC‐5	Waste Water Sewage	Fe	Fe‐N_3_O	Fenton‐like Reaction	High removal efficiency of phenol, 4‐NP, SMX High BPA removal efficiency of 87.5% during a 48‐h operation with 1087 bed volumes.	[66]
	SACo(24)‐N/C	Lignin	Co	Co‐ N_4_	Fenton‐like Reaction	NPX degradation catalytic activity of 0.241 min^−1^ Efficient in degrade other contaminants like PCM, CIP, BPA	[208]
	Fe–N_4‐_C	Enteromorpha	Fe	Fe‐N_4_‐C	Fenton‐like Reaction	Near 100% degradation of NPX in 10 min Stability across a range of pH conditions and different water feedstock	[58]
	PABC‐750	P. americana	Mn	Mn‐N_4_	Photo‐Fenton‐like Reaction	RhB degradation rate of ∼ 100% in 10 min Degradation rate above 90% after 6 cycles	[63]

## 7 Life Cycle Assessment (LCA) of Sus‐SACs

Beyond their near‐100% atom utilization, Sus‐SACs offer additional core advantages, including resource sustainability and waste valorization. Such a comprehensive environmental assessment is essential for enabling the transition of Sus‐SACs from laboratory research to large‐scale industrial application. In this regard, LCA serves as a systematic framework for quantitatively evaluating the environmental impacts of a product or technology across its entire life cycle, encompassing stages from raw material extraction to end‐of‐life disposal.

The methodological principles and analytical framework of LCA have been comprehensively summarized in previous reviews [209]. Briefly, a standard LCA framework consists of four stages, including goal and scope definition, life cycle inventory (LCI), life cycle impact assessment (LCIA), and interpretation [210]. The goal and scope phase requires a clear definition of the functional unit (e.g., the environmental burden per 1 mol of CO_2_ converted) as well as the establishment of system boundaries, including considerations of upstream supply chains and equipment operation. The LCI stage subsequently involves compiling detailed inputs and outputs, including raw material usage, energy consumption, and waste emissions. During LCIA, these inventory flows are characterized into multiple environmental categories, such as global warming potential (GWP), acidification potential (AP), resource depletion, human toxicity, and ecotoxicity, through established methodologies including ReCiPe or CML. Finally, the interpretation phase incorporates sensitivity analysis to examine result robustness and enhance confidence in the overall conclusions [211].

At the raw material extraction stages, conventional SACs depend on the use of high‐purity chemicals while Sus‐SACs relies on the use of biomass or solid waste with greater compositional heterogeneity. This distinction can influence LCA outcomes, as waste‐derived feedstocks may reduce upstream environmental burdens associated with resource extraction while simultaneously introducing additional impacts related to collection, transportation, sorting, and pretreatment. Moreover, composition heterogeneity can increase process complexity and energy demand, thereby contributing to uncertainty in LCI data. Accurate modeling of these trade‐offs is therefore essential for robust LCA.

Moreover, the sustainability of Sus‐SACs is strongly influenced by their synthesis routes. At present, pyrolysis is the dominated synthetic method for Sus‐SACs. However, its typically high operating temperatures and prolonged processing durations can result in substantial energy demand and associated environmental burdens [212]. Developing energy and time‐efficient synthetic methods, including low‐temperature or ultrafast synthesis, is therefore essential to reduce the environmental impact of Sus‐SACs. In addition, the optimization of synthesis parameters is critical to achieve greener synthesis of Sus‐SACs. Such a process can be effectively supported by machine learning techniques.

At the usage stage, the atomically dispersed active sites in Sus‐SACs enhance atomic utilization, thereby potentially reducing both catalyst loading and associated energy requirements. However, the life‐cycle performance of Sus‐SACs is closely tied to their long‐term stability under practical operating conditions. Insufficient durability may necessitate frequent catalyst replacement, thereby increasing operational costs, while metal leaching can introduce additional environmental burdens. Accordingly, the rational design of metal–support interactions to suppress leaching and sintering is critical for improving catalyst stability and minimizing the overall environmental footprint of Sus‐SACs. Additionally, the development of new device designs may also minimize the metal losses during operation. The final stage of the Sus‐SAC life cycle involves catalyst disposal or recycling, yet this phase remains largely overlooked in current research. Inadequate recovery, recycling, or reuse could partially offset the environmental benefits achieved during earlier stages, thereby undermining the overall sustainability of Sus‐SAC systems [213]. Future development of Sus‐SACs should incorporate recyclability and recovery efficiency as key design considerations from the outset.

In a representative study, Zhang and colleagues demonstrated the upcycling of diverse waste sludge into high‐value SACs for Fenton‐type catalysis, coupling process development with integrated LCA and techno‐economic assessments [65]. Results showed that the valorization pathway outperformed direct incineration in terms of both life‐cycle impacts and environmental costs. Sensitivity analysis identified electricity use as the principal driver of environmental burden, prompting the incorporation of forward‐looking grid decarbonization scenarios spanning 2022–2060. Carbon emissions from both treatment routes were found to scale with electricity intensity. Long‐term projections further indicated substantial economic advantages, with potential savings reaching roughly $7.6 billion by 2030 and $15.6 billion by 2060. Importantly, these estimates are likely conservative, given that future improvements in clean production technologies, scale‐driven cost reductions, and rising SAC market prices were not fully captured.

At present, LCA of Sus‐SACs remains relatively limited, with most studies primarily emphasizing catalytic performance characterization. Although the field is still at an early stage of development, LCA will be essential for guiding the transition from laboratory exploration to practical real‐world applications.

## 8 Conclusions and Future Perspectives

The synthesis of SACs from biomass‐ and solid waste‐derived precursors marks an important step forward in the sustainable development of SACs field. In this review, we summarize the diverse precursor sources used for constructing Sus‐SACs and introduce the emerging role of AI technologies in the rapid, non‐destructive characterization and screening of the precursors. Furthermore, we discuss the synthesis strategies, particularly recent advances in ultrafast methods, as well as key characterization techniques. Finally, the recent advances of Sus‐SACs in the application of electrocatalysis, chemical synthesis, and upgrade and environmental remediation are introduced. Despite the significant progress made in this field, several challenges remain, and further developments are needed to fully unlock the potential of Sus‐SACs.

1. As summarized in Table 1, pyrolysis remains the predominant synthesis route for Sus‐SACs, yet its practical limitations are increasingly evident. Most pyrolysis‐derived Sus‐SACs are produced with a yield of milligram scale, far from meeting the material demands for large‐scale deployment. In addition, conventional pyrolysis typically involves multistep procedures that are time‐ and energy‐intensive, thereby constraining its throughput and scalability. These challenges highlight the urgent need for the development of ultrafast or continuous‐flow fabrication approaches that have the potential to substantially reduce processing time and enable the kilogram‐ even to ton‐scale production. Advancing such scalable synthesis technologies will be crucial for bridging the gap between laboratory‐level demonstrations and practical industrial implementation of Sus‐SACs.
2. Most reported Sus‐SACs exhibit relatively low metal loadings, typically below 5 wt.%. Such low loadings pose a technical challenge, as the limited areal density of active sites restricts catalytic productivity and hinders the attainment of high reactivity [118, 214, 215]. Increasing metal loading is therefore essential, particularly for non‐noble metal–based Sus‐SACs, to maximize catalytic performance per unit reactor volume or surface area in large‐scale applications. Achieving this, however, requires the development of supports with a sufficient density of anchoring sites capable of stabilizing isolated metal atoms. Rational modification of Sus‐SACs to enrich defect sites, heteroatom contents, and coordination motifs will be crucial for enabling higher metal dispersion and needs extensive future investigation.
3. AI technologies have shown great promise in accelerating rapid characterization, precursor selection, and structural prediction. Thus, integrating AI into the development of Sus‐SACs holds significant potential for expediting catalyst discovery and optimization. Moreover, given the inherent complexity and variability of biomass‐derived precursors, combining AI with computational modeling can help elucidate structure‐activity relationships that are otherwise difficult to capture. This multidisciplinary research paradigm not only enables efficient screening of high‐performance Sus‐SACs but also reduces experimental workload.
4. In most studies on Sus‐SACs, sustainability is considered primarily from the perspective of using biomass or solid waste as precursors, while the environmental and ecological impacts of the entire synthesis route are often overlooked. In future research, incorporating comprehensive LCA will be essential for evaluating the true sustainability of Sus‐SACs. Although biomass‐ and solid waste‐derived catalysts are generally considered environmentally favorable, their overall ecological footprint, including precursor collection, processing, energy consumption during synthesis, emissions, and end‐of‐life management, remains unexplored. Conducting LCA will enable quantitative comparisons among different synthesis routes. Stages with the highest environmental burden will be identified, and in turn, the design of greener production strategies can be guided. Ultimately, incorporating LCA into Sus‐SAC development will enhance the credibility of sustainability claims and facilitate the transition of Sus‐SACs from laboratory research to practical industrial applications.
5. In recent years, dual single‐atom catalysts with active sites composed of two metal atoms have been reported. The incorporation of diatomic structures breaks the structural uniformity inherent to single‐atom systems by enabling a broader range of reactant binding configurations. The resulting diversity in adsorption and activation sites gives rise to alternative electron‐transfer pathways, thereby expanding the mechanistic possibilities and offering greater flexibility for catalytic reactions [216]. Therefore, developing dual‐metal Sus‐SACs may produce synergistic “1 + 1>2” effects, enabling catalytic performance that surpasses the sum of their individual contributions.

## Conflicts of Interest

The authors declare no conflicts of interest.
